# Evaluation of the high-quality development of graduate education in mining engineering based on ISM and Bayesian networks

**DOI:** 10.1371/journal.pone.0359427

**Published:** 2026-10-01

**Authors:** Liyun Wu, Zhipeng Sun, Yuzhong Yang

**Affiliations:** School of Energy Science and Engineering, Henan Polytechnic University, Jiaozuo, China; Istanbul Technical University: Istanbul Teknik Universitesi, TÜRKIYE

## Abstract

The quality of graduate education in mining engineering is important for safeguarding national energy security and supporting the green and intelligent transformation of the mining industry. This study developed an evaluation system comprising five criterion-level dimensions, 12 sub-criteria, and 26 indicators. Interpretive Structural Modeling (ISM) was used to identify the hierarchical relationships among the indicators, and an expert-informed Bayesian network was subsequently established for probabilistic evaluation. The results showed that the target node had a positive-state probability of 47%, indicating an overall medium level of development. Curriculum quality exhibited the highest positive-state probability among the sub-criteria. When the target node was set to the positive state, the core curriculum renewal cycle, supervision frequency in practical training, and supervisors’ technological updating capability showed comparatively high posterior probabilities, whereas research platform support emerged as a relatively weak node requiring further attention. These findings provide structured decision-support evidence for education administrators and policymakers seeking to improve the quality of graduate education in mining engineering in China.

## 1. Introduction

The concept of high-quality development, first formally introduced at the 19th National Congress of the Communist Party of China [1,2], marked China’s transition from a phase of rapid growth to a new stage of development centered on quality. As a vital component of the national innovation system and talent development system, higher education has entered a new stage of development centered on quality enhancement, as China’s blueprint for building a strong education system increasingly prioritizes quality, equity, reform, and openness [3–5]. Graduate education, positioned at the apex of the higher education system, bears the critical mission of cultivating top-tier innovative talent and advancing scientific and technological innovation, and is increasingly shaping the development trajectory of higher education [6,7]. With the continued upgrading of China’s economic structure and the deepening implementation of the innovation-driven development strategy, graduate education has assumed the vital responsibility of providing high-quality talent support for major national strategic needs and key industrial sectors [8].

China’s energy resource endowment is characterized by an abundance of coal, limited oil, and scarce natural gas [9,10], with proven coal reserves accounting for 33.8% of the world’s total coal reserves. In 2025, China’s raw coal output reached 4.85 billion tons, and its coal production has ranked first in the world for many consecutive years. Coal has long occupied a dominant position in China’s primary energy structure, accounting for more than 90% of total energy consumption in the 1950s [11]. In recent years, although the growth rate of coal consumption has slowed and its share in the overall energy consumption mix has continued to decline, it still remained as high as 51.4% in 2025, indicating that coal will continue to serve as a fundamental energy source for safeguarding national energy security over the long term [12]. Graduate education in mining engineering serves as the primary platform for cultivating high-level technical and managerial talent required for coal resource development, safe production, green mining, and intelligent transformation. Its development quality therefore bears directly on the high-quality transformation of the mining industry and holds substantial practical significance for safeguarding national energy security.

Although this study focuses on graduate education in mining engineering in China, the coordination between graduate training systems and the demands of mining-industry transformation is not unique to China. The global energy transition, the development of sustainable mining, and the application of digital and automated technologies are reshaping the knowledge structures and competency requirements of mining occupations, placing greater demands on mining professionals’ digital competencies, interdisciplinary collaboration capabilities, environmental governance capabilities, and ability to solve complex engineering problems. Therefore, although the evaluation framework developed in this study is based on the Chinese context, it may provide a methodological reference for other countries and regions undergoing mining-industry transformation to evaluate the quality of graduate education and identify weaknesses in their training systems.

Existing studies have proposed various frameworks for evaluating graduate education quality, primarily focusing on the construction of indicator systems and weighting methods, such as the Analytic Hierarchy Process (AHP) [13], Particle Swarm Optimization (PSO) [14], and expert-based scoring models [15]. Although these methods provide useful insights into the relative importance of different indicators, they still have certain limitations [16–18]. First, they lack discipline-specific analysis, particularly for fields such as mining engineering that are strongly embedded in industrial contexts. These approaches have limited capacity to explain how curriculum design, faculty support, practical training, research platforms, and industry needs interact to shape the high-quality development of graduate education. Moreover, most existing evaluation frameworks do not adequately capture the strong industrial embeddedness of mining engineering. In this discipline, talent development is closely linked to mine safety, intelligent mining, and national energy security. Second, they tend to treat evaluation indicators as independent variables, failing to capture the hierarchical and causal relationships among them. Finally, they rarely incorporate uncertainty into the evaluation process, which limits their ability to model real-world educational systems. Overall, existing studies have generated valuable insights into the conceptualization of the high-quality development of graduate education and the construction of general evaluation frameworks; however, they remain insufficient in terms of industry specificity, methodological systematicity, and causal mechanism analysis. Compared with previous studies, this study develops a discipline-specific, mechanism-oriented evaluation framework tailored to graduate education in mining engineering. First, the indicator system is designed around the distinctive demands arising from the transformation of the mining industry, with particular emphasis on green mining and intelligent mining rather than on generic indicators of educational quality. Second, Interpretive Structural Modeling (ISM) is used to identify the hierarchical influence structure among the indicators and to transform a static evaluation index system into a structured hierarchy. Third, the relationships identified through ISM are incorporated into a Bayesian network, enabling probabilistic inference under uncertainty and supporting both forward and diagnostic inference. Accordingly, this study takes graduate education in mining engineering as the research object and constructs an ISM-based evaluation index system together with a Bayesian network-based evaluation model, with the aim of systematically identifying the key influencing factors and clarifying the causal mechanisms underlying its high-quality development.

The main contributions of this study are as follows:

(1) A discipline-specific evaluation framework was developed for the high-quality development of graduate education in mining engineering. The framework incorporates indicators that reflect the distinctive needs of the discipline, including intelligent geoscience-and-mining courses, green mining courses, mine-safety practice, and the alignment of thesis topics with industry needs. It thereby extends the evaluation of graduate education from a generic quality-assessment perspective to one that emphasizes industrial embeddedness and transformation-oriented development.
(2) By integrating ISM with a Bayesian network, this study advances the methodological framework for evaluating graduate education. ISM is used to identify the hierarchical influence relationships among the indicators, while the Bayesian network translates these structured relationships into a probabilistic reasoning framework. This integrated approach overcomes the limitations of conventional static weighting and linear aggregation methods by representing probabilistic dependencies, hierarchical propagation, and uncertainty among multiple educational factors.
(3) Through forward inference, diagnostic inference, and sensitivity analysis within the Bayesian network, this study identifies the key factors associated with the high-quality development of graduate education in mining engineering and examines their potential influence pathways. It further clarifies the distinct roles of the training process and educational environment within the overall evaluation system.

## 2. Literature review

### 2.1. The connotation of high-quality development in graduate education

Graduate education quality has long been a central topic in higher education research. Early studies primarily focused on the relationship between educational resources and outcomes, emphasizing such aspects as student quality, faculty resources, training conditions, and academic achievements. With the evolution of educational concepts and the introduction of the notion of “high-quality development,” increasing attention has been paid to innovation capacity, effectiveness, sustainability, and alignment with societal needs. Wang and Zhou examined how high-quality graduate education can be advanced through the optimization of training objectives, training processes, and training approaches, thereby promoting the high-quality development of higher education [19]. Linde and Petrova argue that high-quality graduate education should focus on the continuous enhancement of students’ research and innovation capabilities, aiming to cultivate high-level talents with strong social adaptability and an international perspective [20]. Hagenauer et al. investigated higher education teachers’ perceptions of teacher–student relationships and their influencing factors, finding that the connotation of high-quality development in graduate education involves not only professional support and academic interaction, but also the interpersonal and emotional dimensions of teacher–student relationships [21]. In summary, the connotation of high-quality development extends beyond the improvement of academic outcomes; it also reflects the deep integration of educational models, faculty capacity, societal needs, and industrial development, emphasizing the close alignment between education and socio-economic demands, as well as the innovation and sustainability of talent cultivation.

### 2.2. Evaluation system for the high-quality development of graduate education

Current discussions on evaluation index systems for high-quality development have mainly focused on the economic field, while relatively limited attention has been paid to evaluation systems for the high-quality development of higher education. In response, some scholars have constructed multidimensional evaluation index systems based on multi-source data, covering such dimensions as educational foundations, resource input, training processes, development performance, and social support, and have applied the AHP and the Natural Breaks Method for comprehensive evaluation [22–25]. In addition, Wu and Lin drawing on the concept of Outcome-Based Education (OBE), developed an evaluation index system for teaching quality management in application-oriented universities. The system comprises six first-level indicators and fifteen second-level indicators, emphasizing a student-centered approach as well as the balanced development of process and competence, and systematically addressing the problems of missing indicator systems and content bias in evaluating the high-quality development of higher education [26]. Tang et al. based on the CIPP model and the AHP-FCE method, explored multidimensional indicators for educational quality evaluation, providing an effective assessment tool for entrepreneurship education in higher education institutions [27]. Ji et al. constructed an evaluation index system encompassing dimensions of quality transformation, efficiency transformation, and transformation, and applied the entropy weight method and the TOPSIS model for comprehensive assessment. Their findings indicate that the evaluation of high-quality development in graduate education should emphasize the synergy among educational outcomes, resource support, and development potential, taking into account both training processes and outcomes, while also considering regional disparities and the role of human capital [28]. Goel et al. applied ISM and the AHP to green human resource management in educational institutions. ISM was used to identify the interrelationships and hierarchical structure among implementation challenges, while AHP was employed to prioritize these challenges and validate the results. Their findings demonstrate that this integrated approach can provide structured analysis and decision support for complex educational management problems [29]. In addition, to address the ambiguity and uncertainty inherent in educational decision-making, Al-Gerafi et al. developed an interval-valued fuzzy multi-criteria decision-making model, providing a methodological reference for the comprehensive evaluation and prioritization of educational alternatives under uncertain information [30].

Overall, existing studies have provided important references for evaluating the high-quality development of higher education in terms of the construction of evaluation dimensions, the selection of methods, and the expansion of application scenarios. However, although most existing evaluation index systems cover multiple dimensions, they remain largely based on the static weighting of indicators and lack a systematic representation of the complex relationships and hierarchical structures among variables, making it difficult to reveal the dynamic mechanisms through which different factors interact. In addition, existing studies have largely focused on macro-level evaluations at the regional or institutional level, while relatively insufficient attention has been paid to the characteristics of talent cultivation in mining engineering and its strong industrial embeddedness. Therefore, this study focuses on the high-quality development of graduate education in mining engineering and attempts to construct an evaluation framework that integrates hierarchical structure, system complexity, and dynamic analysis. The aim is to more accurately identify key influencing factors and their interaction pathways, thereby providing both theoretical support and practical guidance for improving the quality of graduate education in industry-specific disciplines.

### 2.3. Conceptual framework for the high-quality development of graduate education in mining engineering

The high-quality development of graduate education in mining engineering should be understood as a systemic process shaped by the interaction among national education strategies, the demands of industrial transformation, and discipline-specific training mechanisms. From the perspective of high-quality development, graduate education is no longer evaluated solely on the basis of conventional scale-oriented indicators, such as enrollment size, the number of courses, or research output. Greater emphasis is instead placed on the quality of talent development, innovation and practical capabilities, adaptability to industrial transformation, and the sustainable contribution of education to socioeconomic development. Accordingly, the high-quality development of graduate education in mining engineering is conceptualized in this study as a formal multidimensional formative construct comprising five interrelated dimensions: training foundation, support capacity, process control, outcome transformation, and industry adaptability. Training foundation reflects the quality of the curriculum system, practical training, and thesis-topic design. Support capacity encompasses the roles of faculty, research platforms, management systems, and incentive mechanisms. Process control emphasizes the monitoring of practical training, thesis quality, and enterprise feedback. Outcome transformation concerns the translation of academic achievements into papers, patents, technologies, and practical applications. Industry adaptability reflects the extent to which graduates, training programs, and specialized training tracks respond to the evolving needs of the mining industry. These dimensions and their corresponding indicators do not operate independently; rather, they jointly form a hierarchical and interdependent system. For example, research platforms, faculty capacity, and management mechanisms provide underlying support for curriculum renewal, practical training, and process monitoring, which are in turn associated with academic outcomes, technology transfer, and employment adaptability.

The conceptual model developed in this study follows the logic of “strategic demands–disciplinary responses–educational processes–development outcomes.” The national high-quality development agenda and the transformation of the mining industry constitute the external strategic demands, while green mining, intelligent mining, mine safety, and industry–education integration represent the discipline-specific responses required of graduate education in mining engineering. These responses are progressively translated into internal training processes, including curriculum development, practical training, faculty development, platform support, thesis management, and enterprise feedback. These training processes are, in turn, associated with development outcomes such as high-level academic achievements, technology transfer, graduates’ employment adaptability, and the responsiveness of training programs to industry needs. This conceptual model provides a theoretical foundation for constructing the evaluation index system and for subsequently applying ISM and a Bayesian network to analyze hierarchical influence relationships, probabilistic dependencies, and uncertainty.

## 3. Methodology

### 3.1. Data collection and analysis

This study is guided by the core connotation of the high-quality development of graduate education, and integrates the emerging demand for high-level talent driven by the intelligent transformation of the geoscience and mining industry and the green development goals under the “dual carbon” strategy. A three-stage research approach—comprising literature review, multi-stakeholder interviews, and questionnaire surveys—is employed to systematically identify the key factors underpinning the high-quality development of graduate education in mining engineering, thereby ensuring the scientific rigor and comprehensiveness of the study.

First, national policy documents and education reports related to high-quality development were reviewed, including the Opinions on Accelerating the Reform and Development of Graduate Education in the New Era, the Action Plan for the High-Quality Development of Graduate Education (2024–2027), and the China Graduate Education Quality Report. Factors were included if they were directly related to graduate education quality, talent development, research training, industry needs, green mining, intelligent mining, mine safety, or industry–education integration. Factors were excluded if they were primarily scale-oriented, substantially overlapped with other factors, or lacked sufficient relevance to mining engineering. This process yielded an initial set of 128 potential factors related to the high-quality development of graduate education. Second, participants were selected from universities, mining enterprises, and industry associations. The university group comprised 20 doctoral supervisors in geoscience- and mining-related disciplines and 10 graduate education administrators, with interviews focusing on how curricula and practical training could improve the quality of talent development. The enterprise group included 15 technical professionals and eight human resource managers from mining companies, with interviews focusing on the core competencies required of mining professionals. The industry-association group comprised six experts from the China National Coal Association and four representatives from the Henan Coal Society, with interviews addressing the emerging requirements of industrial transformation for graduate education. The interviews focused on the gaps between current graduate training and the requirements of high-quality development, the key conditions supporting such development, and the criteria for evaluating graduate education in mining engineering. All interview records were organized and analyzed using thematic coding. In the first round, open coding was conducted to identify concepts and statements related to graduate education quality, mining-industry transformation, green mining, intelligent mining, and industry–education integration. In the second round, similar codes were merged, while repetitive, ambiguous, or weakly relevant codes were removed. In the third round, the remaining codes were grouped into higher-order categories, including curriculum quality, practical training, faculty capacity, platform resources, process control, outcome transformation, and industry adaptability. To enhance the credibility of the coding process, two researchers independently reviewed and coded the interview materials and subsequently compared their results. Discrepancies were resolved through discussion, and codes were revised when necessary according to the research focus, indicator relevance, and operationalizability until consensus was reached. Through this process, the initial 128 factors were consolidated into 68 candidate indicators. A questionnaire entitled Influencing Factors in the High-Quality Development of Graduate Education in Geoscience- and Mining-Related Disciplines was then developed on the basis of the 68 candidate indicators. Its content reflected key aspects of the construct, including innovation capacity, quality standards, industry adaptability, and sustainability. A five-point Likert scale was used, ranging from 1 (“not important at all”) to 5 (“extremely important”). A total of 800 questionnaires were distributed across 28 Chinese universities offering geoscience- and mining-related programs, including China University of Mining and Technology and Henan Polytechnic University. Of these, 692 valid questionnaires were retained, yielding a valid response rate of 86.5%. Questionnaires with incomplete responses or evident response-quality problems were excluded. The valid responses were used to further screen the 68 candidate indicators according to their importance, disciplinary relevance, feasibility, redundancy, and operational measurability. Ultimately, 32 indicators were retained for subsequent expert validation and refinement. It should be noted that, before conducting the interviews, expert consultations, and questionnaire survey, the researchers informed all participants of the study objectives, the nature of their participation, the intended use of the data, the anonymization procedures, the confidentiality measures, and the voluntary nature of participation. After fully understanding the relevant information, all participants provided verbal informed consent. The researchers responsible for data collection documented the participants’ informed consent in the data collection records and proceeded with subsequent data collection only after confirming their consent.

### 3.2. Determination of the final evaluation indicators

The construction of the evaluation index system was guided by the overarching objective of high-quality development and followed five principles. First, under the quality-orientation principle, indicators were required to directly reflect the objectives of high-quality development in graduate education rather than merely capture conventional quantitative expansion. Examples include the proportion of granted invention patents meeting predefined quality criteria and the employment rate of graduates in relevant mining-industry positions. Second, under the scientific rigor principle, each indicator was required to be theoretically grounded, clearly defined, and measurable. For example, the quality level of practical training bases can be measured as the proportion of national-level or otherwise high-level bases among all practical training bases, thereby avoiding vague or difficult-to-measure indicator definitions. Third, under the systematicity principle, the indicators were designed to cover the entire chain of training foundation, support capacity, process control, outcome transformation, and industry adaptability, while maintaining clear logical connections. For instance, supervisors’ technological updating capability is linked to the proportion of intelligent geoscience-and-mining courses, which is further associated with outcome transformation. Fourth, under the discipline-specificity principle, the indicators were tailored to the transformation needs of the geoscience and mining sector, as reflected in indicators such as mine-safety practice coverage and the proportion of green mining courses. Fifth, under the operational feasibility principle, the required data had to be readily obtainable. For example, the dual-supervisor allocation rate can be calculated from graduate education management systems. On the basis of these principles, the final evaluation index system was developed through the following four steps.

The first step involved an initial review of the 32 indicators retained from the preceding screening stage. The indicators were examined in terms of their alignment with high-quality development, disciplinary relevance, measurability, and suitability for model construction. Indicators that were weakly related to the construct or primarily reflected general competencies were excluded. For example, although graduate students’ English proficiency may reflect general learning ability, it is not directly aligned with the specific objectives of the high-quality development of graduate education in mining engineering and was therefore excluded from the final system. The second step involved expert validation using the Delphi method. Twenty experts rated the necessity of each indicator for supporting high-quality development on a five-point scale. Indicators scoring below 4, such as the number of international conferences attended by graduate students, were removed because of their limited relevance to the quality requirements of geoscience- and mining-related education, leaving 28 indicators. The third step involved Pearson correlation analysis of the survey data for the 28 indicators. For indicator pairs with correlation coefficients greater than 0.85, decisions were made on the basis of conceptual relevance and explanatory value, with priority given to indicators that more directly represented the construct. For example, the number of practical training bases was highly correlated with the quality level of those bases; because the latter better captured the quality of the practical training platform, it was retained, while the former was removed. This step reduced the indicator set to 24. The fourth step involved content validation against the core requirements of the high-quality development of graduate education in mining engineering, with particular attention to green mining, intelligent mining, mine safety, and industry–education integration. The review indicated that the existing set did not adequately represent green mining curriculum development or mine-safety practice. Accordingly, the proportion of green mining courses and mine-safety practice coverage were added. The final evaluation system therefore comprised 26 indicators, which served as the basis for subsequent ISM and Bayesian network modeling.

### 3.3. ISM model

#### 3.3.1. Construction of the ISM model.

ISM is a systems-structuring method [31] that has been widely applied in complex-system analysis, decision support, and sustainability research. It is particularly useful for identifying hierarchical relationships among interacting factors [32]. Based on the 26 indicators, this study constructed a four-level evaluation index system comprising the target level, criterion level, sub-criterion level, and indicator level, with the aim of clarifying the hierarchical relationships among the indicators and identifying the underlying structural drivers. The specific construction procedure is described below.

The first step was to determine the indicator set and define the corresponding symbols. The 26 indicators were coded and specified, with the criterion level corresponding to five high-quality dimensions (A_1_-A_5_), the sub-criterion level corresponding to 12 high-quality sub-objectives (B_1_-B_12_), and the indicator level corresponding to 26 high-quality indicators (C_1_-C_26_).

The second step was to construct the adjacency matrix. Fifteen experts—including five heads of geoscience and mining-related academic programs, five senior technical executives from enterprises, and five specialists in educational evaluation—were invited to assess the direct influence relationships among the 26 indicators. If indicator C_i_ was considered to exert a direct influence on C_j_, the corresponding entry was assigned a value of 1; otherwise, it was assigned 0, thereby forming the adjacency matrix A (26 × 26). For example, because C_10_ directly influences C_6_, A (10, 6) = 1; because C_5_ directly influences C_7_, A (5, 7) = 1; and because C_22_ does not directly influence C_12_, A (22, 12) = 0.

The third step was to calculate the reachability matrix. Based on the adjacency matrix A, the reachability matrix M was derived using the formula M = (A + I)^k^, where I denotes the identity matrix and k the number of iterations, until (A + I)^k^ = (A + I)^k+1^. The resulting reachability matrix M captures both the direct and indirect influence relationships among the indicators. For example, in the reachability matrix M, M (10, 7) = 1 indicates that C_10_ indirectly influences C_7_ through C_6_, whereas M (5, 22) = 1, indicates that C_5_ indirectly influences C_22_ through C_8_.

The fourth step was to determine the hierarchical structure of the indicators. First, the reachability set and antecedent set were defined. For each indicator C_*i*_, the reachability set R(C_*i*_) denotes the set of indicators influenced by C_i_, that is, R(C_i_) = {C_j_ | M (i, j) = 1}, whereas the antecedent set Q(C_i_) denotes the set of indicators influencing C_i_, that is, Q(C_i_) = {C_j_ | M (j, i) = 1}. Second, the top-level indicators (Level 1) were identified. If R(C_i_) ∩ Q(C_i_) = R(C_i_), then C_i_ was classified as a top-level indicator, indicating that it does not further influence other indicators within the hierarchy. Based on this criterion, the Level 1 indicators were determined to be C_23_, C_24_, and C_26_. Finally, the top-level indicators were removed and the procedure was repeated iteratively: the rows and columns corresponding to the Level 1 indicators were deleted, and the reachability and antecedent sets of the remaining indicators were recalculated to identify the Level 2 indicators (C_20_, C_21_, C_22_, C_25_). This process was repeated until all indicators were assigned to a hierarchy, ultimately yielding a six-level structure. As shown in Table 1, detailed definitions and attributes of the indicators are provided in S1 Table.

**Table 1:** ISM hierarchical structuring.

Level	Indicator code	Hierarchical attribute
1	C_23_、C_24_、C_26_	Development outcome indicators
2	C_20_、C_21_、C_22_、C_25_	Intermediate outcome indicators
3	C_8_、C_16_、C_17_、C_18_	Process control indicators
4	C_6_、C_7_、C_9_、C_19_	Practical support indicators
5	C_1_、C_2_、C_3_、C_4_、C_5_、C_10_	Foundational support indicators
6	C_11_、C_12_、C_13_、C_14_、C_15_	Core driving indicators

Based on the hierarchical partitioning results, the structural relationships between underlying drivers and development outcomes can be summarized as follows. The core driving layer comprises five indicators and represents the deepest structural drivers in the ISM hierarchy. The foundational support layer contains six indicators, including the proportion of intelligent geoscience-and-mining courses and the quality level of practical training bases, and provides the basic conditions for the overall evaluation system. The practical support layer consists of four indicators, including the dual-supervisor allocation rate and the duration of field practice, and connects the foundational support layer with the process control layer. The process control layer includes four indicators, such as thesis-process control and the response speed of enterprise feedback, and is closely associated with the quality of subsequent outcomes. The intermediate outcome layer comprises four indicators, including the proportion of high-level papers and the thesis-outcome transformation rate, and reflects the intermediate manifestations of development. The development outcome layer contains three indicators, including the value of technology transfer and the employment rate of graduates in relevant mining-industry positions, and represents the final outcomes of the evaluation system.

As shown in Table 1, the Level 6 indicators, including Supervisors’ technological updating capability and Research platform support, constitute the primary leverage points for promoting high-quality development and therefore warrant priority investment. The four Level 4 indicators, including the Dual-supervisor allocation rate and the duration of field practice, directly connect the foundational support layer with the process control layer and thus should be further strengthened in implementation. In addition, outcome-oriented indicators require balanced attention: both development outcome indicators and intermediate outcome indicators should be improved in a coordinated manner to avoid an overemphasis on academic output at the expense of practical application.

#### 3.3.2. ISM-Based evaluation index system.

Based on the above ISM-based hierarchical analysis, a four-level coordinated evaluation index system for the high-quality development of graduate education in mining engineering was constructed, as shown in Table 2. Detailed definitions and attributes of the indicators are provided in S1 Table.

**Table 2:** Evaluation index system for the high-quality development of graduate education in mining engineering.

Target level (O)	Criterion level (A, 5 dimensions)	Sub-criterion level (B, 12 sub-criteria)	Indicator level (C, 26 indicators)
High-quality development level of graduate education in mining engineering	A_1_: Training foundation	B_1_: Curriculum quality	C_1_-C_4_
		B_2_: Practical training	C_5_-C_7_
		B_3_: Thesis quality	C_8_
	A_2_: Support capacity	B_4_: Faculty capacity	C_9_-C_11_
		B_5_: Platform resources	C_12_-C_13_
		B_6_: Management system	C_14_-C_15_
	A_3_: Process control	B_7_: Process monitoring	C_16_-C_17_
		B_8_: Feedback mechanism	C_18_-C_19_
	A_4_: Outcome transformation	B_9_: Academic outcomes	C_20_-C_21_
		B_10_: Applied outcomes	C_22_-C_23_
	A_5_: Industry adaptability	B_11_: Job adaptability	C_24_
		B_12_: Responsiveness to industry needs	C_25_-C_26_

As shown in Table 2, the evaluation index system for the high-quality development of graduate education in mining engineering comprises four levels: the target level, criterion level, sub-criterion level, and indicator level. The criterion level includes five operational dimensions: training foundation, support capacity, process control, outcome transformation, and industry adaptability. Each dimension is further specified through the sub-criterion level, which captures key components of educational quality, including curriculum quality, practical training, thesis quality, faculty capacity, platform resources, process monitoring, feedback mechanisms, academic outcomes, applied outcomes, job adaptability, and responsiveness to industry needs. At the indicator level, each sub-criterion is represented by specific measurable indicators. Together, these indicators cover curriculum development, practical training, faculty development, platform support, process management, outcome transformation, and industry responsiveness, thereby providing a structured basis for evaluating graduate education in mining engineering. This multilevel system enables a more detailed assessment of educational quality and supports evidence-informed educational management, policy formulation, and continuous improvement.

### 3.4. Bayesian networks

A Bayesian network [33] is a mathematical model based on probabilistic inference. As a probabilistic structure represented by a directed acyclic graph (DAG), it provides a powerful tool for risk assessment and decision support. A Bayesian network consists of three components: nodes, directed arcs, and conditional probability tables. The nodes represent random variables, the directed arcs denote the conditional dependencies among these variables, and the conditional probability tables specify the type and strength of the conditional dependencies between nodes. In the process of advancing the high-quality development of graduate education in mining engineering, substantial uncertainty arises from multiple interacting factors, including industrial transformation, policy adjustment, and the coupling of training components. Bayesian networks are well suited to handling such uncertainty through probabilistic inference. Their topological structure provides a graphical representation of the relationships among nodes, and enables the modeling of dynamic probabilities in the high-quality development process of graduate education in mining engineering through causal inference, thereby simulating the causal relationships among events. In this study, the Bayesian network is employed as an expert-informed probabilistic reasoning framework that integrates expert knowledge with the theoretical structure identified through ISM to support educational decision-making.

#### 3.4.1. Bayesian network structural.

Based on the preceding ISM-based hierarchical analysis, an evaluation index system for the high-quality development of graduate education in mining engineering was established. Because the nodes in a Bayesian network exhibit a logical structure similar to that of the evaluation index system, the indicators in the evaluation framework can be directly mapped onto the nodes of the Bayesian network. Fig 1 illustrates the transformation from the evaluation index system to the Bayesian network structure. In this structure, the node pointed to by an arrow represents the result, whereas the node at the tail of the arrow represents the cause. For example, B_1_, B_2_, and B_3_ are causal parent nodes of A_1_ (Training foundation).

**Fig 1:**
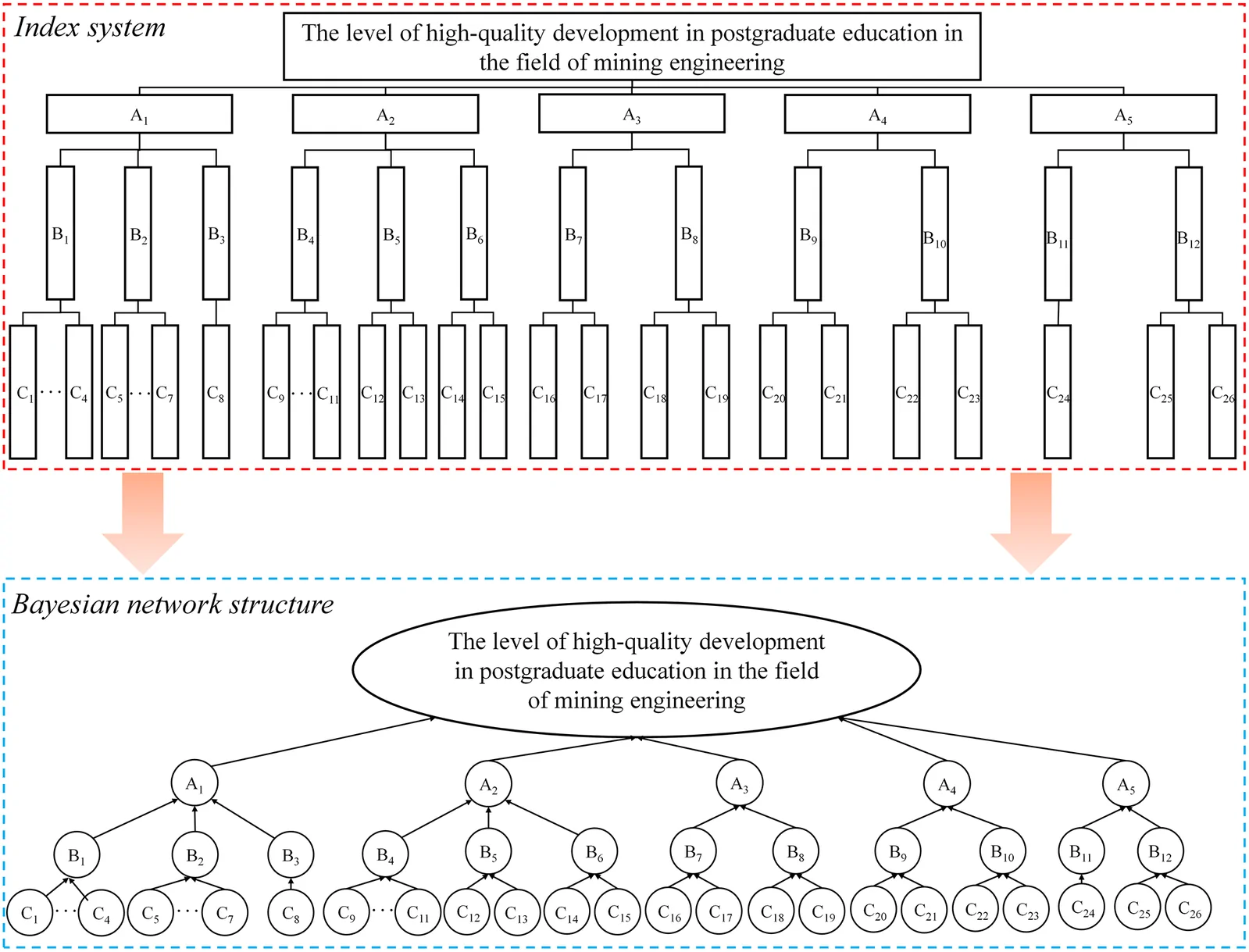
Evaluation-system transformation into a Bayesian network.

Based on the hierarchical influence relationships identified through ISM, the target, criterion, sub-criterion, and indicator levels of the evaluation index system were mapped onto the corresponding nodes of the Bayesian network. Directed connections among the nodes were then established according to the influence directions specified by the ISM structure. The resulting Bayesian network topology was implemented using GeNIe Modeler, as shown in Fig 2.

**Fig 2:**
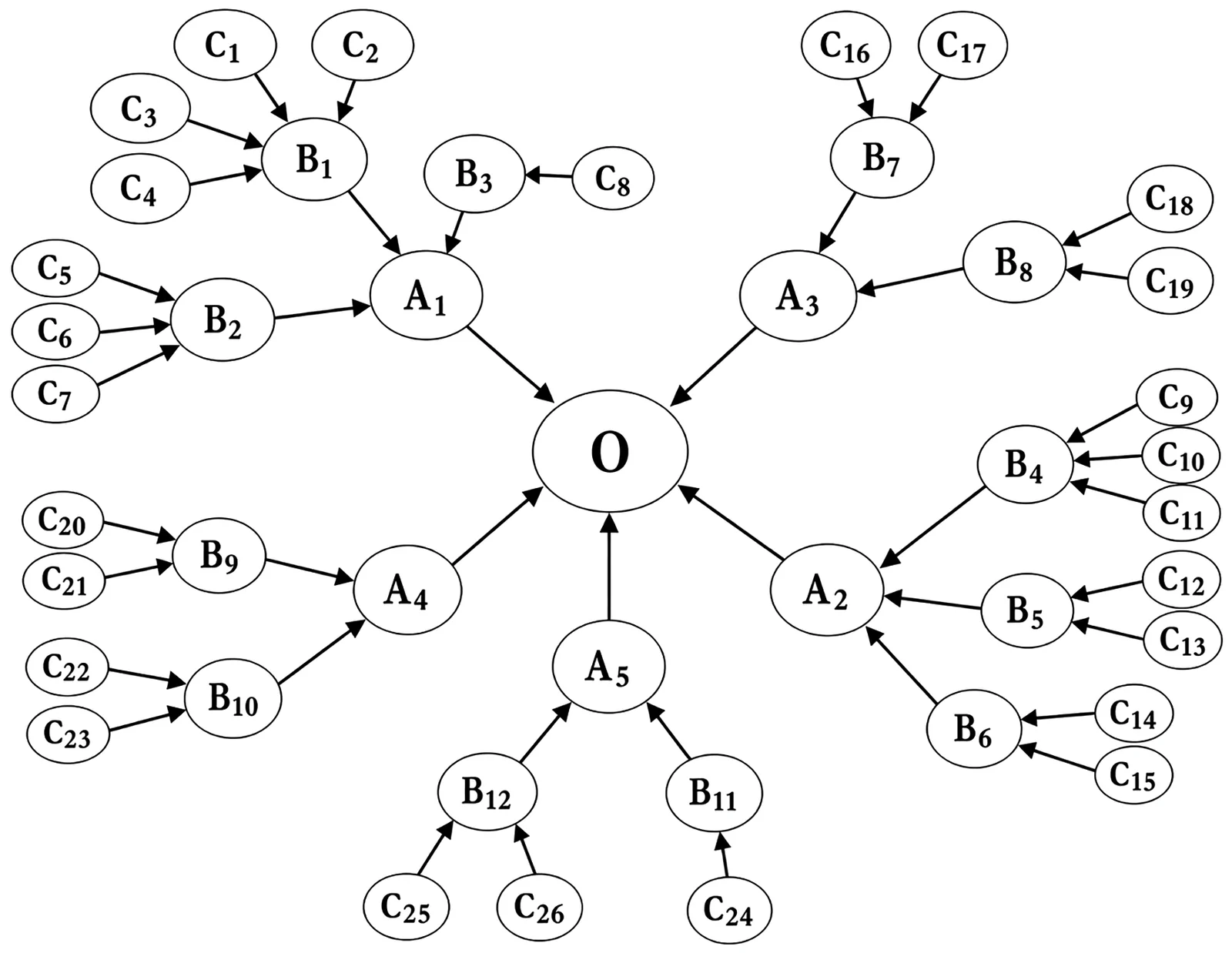
Bayesian topology for mining graduate education development.

#### 3.4.2. Determination of network node states and prior probabilities.

After the Bayesian network topology was established, the states of the nodes and the value domains of the corresponding variables were defined. Each node was assigned two states: Y (Yes), indicating that the educational condition, training process, or development outcome represented by the node met the predefined quality requirement, and N (No), indicating that the corresponding requirement had not yet been met. Accordingly, the state space of each node was defined as {Y, N}, with state probabilities ranging from 0 to 1. For each node C_i_, the two state probabilities were denoted by P (C_i_ = Y) and P (C_i_ = N), satisfying P (C_i_ = N) = 1-P (C_i_ = Y). The Y/N states were not obtained by mechanically dichotomizing the preceding five-point Likert-scale responses using a uniform numerical threshold. The Likert-scale questionnaire was used primarily to assess the importance of candidate factors and to support indicator screening, whereas the Y/N states in the Bayesian network were used to define the probabilistic semantics of the model variables.

For quantitative indicators with observed values, a probability-interval mapping method was applied according to their respective attributes. Let *q* denote the observed value of indicator C_i_, (e, f) its value interval, and (g, h) the corresponding probability interval. Depending on whether the indicator was positively or negatively associated with the high-quality development construct, its observed value was linearly mapped to the probability that the node was in state Y, denoted by P(C_i_ = Y), as shown in Equation (1).


$$\begin{array}{c} \text{P}\left({\text{C}}_{\text{i}}\text{=Y}\right)=\left\{ \begin{array}{c} @c@\frac{\left(\text{q-e}\right)\left(\text{h-g}\right)}{\text{f-e}}\text{+g,}Positive\;correlation\;between\;the\;indicator\;and\;probability\\ \text{h-}\frac{\left(\text{q-e}\right)\left(\text{h-g}\right)}{\text{f-e}},Negative\;correlation\;between\;the\;indicator\;and\;probability \end{array}\right. \end{array}$$ (1)

The probability-interval mapping method does not assign quantitative indicators deterministically to state Y or state N. Instead, state probabilities are used to describe the extent to which an indicator meets the predefined quality requirement, thereby preserving uncertainty in the evaluation process. In addition, to facilitate the qualitative interpretation of node probabilities obtained through Bayesian network inference, three development categories—high, medium, and low—were established. These categories serve only as interpretive criteria for the inference results. They are not used to define the Y/N states of the network nodes and do not enter the calculation of prior probabilities, conditional probability tables (CPTs), or posterior probabilities. The corresponding probability intervals are presented in Table 3.

**Table 3:** Interpretation criteria for development levels based on Bayesian network inference.

Positive-state probability	66% ~ 100%	33% ~ 66%	0 ~ 33%
Evaluation grade	1	2	3
Category	High	Medium	Low

#### 3.4.3. Conditional probabilities.

In a Bayesian network, each child node is associated with a CPT, which characterizes the conditional probability distribution of the child node under different combinations of values taken by its parent nodes. The methods for determining node CPTs generally fall into two categories: the expert knowledge method and the parameter learning method. Given the characteristics of the research object in this study, the expert knowledge method was adopted to determine the CPTs of the nodes in the Bayesian network. According to the principles of Bayesian networks, the AHP was used to calculate indicator weights, and a weight-based conditional probability calculation method was then employed. The specific procedure is as follows:

Suppose that a child node A has multiple parent nodes, denoted by X_1_, X_2_, …, X_r_, with corresponding weights W_1_, W_2_, …, W_r_, satisfying W_1_ + W_2_ + … + W_r_ = 1. The possible states of each node are denoted by a_1_, a_2_, …, a_v_, respectively. Then, the following relationship holds:


$$\begin{array}{c} \begin{array}{c} P\left(A=a_{\text{1}}|X_{\text{1}}=a_{\text{1}},X_{2\cdots r}\neq a_{\text{1}}\right)=W_{\text{1}}\\ P\left(A=a_{\text{1}}|X_{\text{1}}=a_{\text{1}},X_{\text{2}}=a_{\text{1}},X_{3\cdots r}\neq a_{\text{1}}\right)=W_{\text{1}}+W_{\text{2}}\\ \vdots \\ P\left(A=a_{v}|X_{\text{1}}=a_{r},X_{2\cdots r}\neq a_{v}\right)=W_{\text{1}}\\ P\left(A=a_{v}|X_{1\cdots r}=a_{v}\right)=W_{\text{1}}+W_{\text{2}}+\cdots +W_{r}\\ P\left(A=a_{v}|X_{1\cdots r}\neq a_{v}\right)=0 \end{array} \end{array}$$ (2)

The weights were determined using AHP. Experts were invited, through a questionnaire survey, to conduct pairwise comparisons and assign scores to the factors at each hierarchical level. In this study, the 1–9 scale was adopted to score the relative importance of paired factors. Let the two factors being compared be denoted as i and j, the scale values and their interpretations are presented in Table 4.

**Table 4:** Scale assignment and interpretation of the judgment matrix.

Scale	Meaning
a_ij_ = 1	Elements i and j are equally important
a_ij_ = 3	Element i is moderately more important than element j
a_ij_ = 5	Element i is strongly more important than element j
a_ij_ = 7	Element i is very strongly more important than element j
a_ij_ = 9	Element i is extremely more important than element j
a_ij_ = 2,4,6,8	Intermediate values between two adjacent judgments
Reciprocal	If the relative importance of element i over element j is a_ij_, then the relative importance of element j over element i is a_ji_ = 1/a_ij_

According to the evaluation index system, a hierarchical pairwise comparison judgment matrix A_n×n_ was constructed as follows:


$$\begin{array}{c} A_{n\times n}=\left[\begin{array}{cccc} @cccc@a_{11} & a_{12} & \cdots & a_{1n}\\ a_{21} & a_{22} & \cdots & a_{2n}\\ \cdots & \cdots & \cdots & \cdots \\ a_{n1} & a_{n2} & \cdots & a_{nn} \end{array}\right] \end{array}$$ (3)

The expert judgment matrices were then aggregated using the geometric mean method. Specifically, for m experts (m = 1, 2,..., k), the corresponding pairwise comparison matrices were multiplied element by element, and the m – th root was subsequently taken to obtain the integrated judgment matrix I_n×n_, as follows:


$$\begin{array}{c} I_{n\times n}={\left(\prod_{k=1}^{m}a_{ij}^{k}\right)}^{\frac{\text{1}}{m}}=\left[\begin{array}{cccc} @cccc@b_{11} & b_{12} & \cdots & b_{1n}\\ b_{21} & b_{22} & \cdots & b_{2n}\\ \cdots & \cdots & \cdots & \\ b_{n1} & b_{b2} & \cdots & b_{nn} \end{array}\right] \end{array}$$ (4)

Weight calculation is the core step of the AHP. In this study, the geometric mean method was used to calculate the weights. First, the elements in each of the n row vectors of the integrated judgment matrix I were multiplied, and the resulting product for the i-th ow was denoted by K_i_. The formula is given as follows:


$$\begin{array}{c} K_{i}=\prod_{i=1}^{n}b_{i} \end{array}$$ (5)

Next, the n-th root of each component of the new vector Ki was taken to obtain the eigenvector M_i_. The corresponding formula is as follows:


$$\begin{array}{c} M_{i}=\sqrt[{n}]{K_{i}} \end{array}$$ (6)

The eigenvector M_i_ was then normalized to obtain the weight vector W_i_:


$$\begin{array}{c} W_{i}=\frac{M_{i}}{\sum_{i=1}^{n}M_{i}} \end{array}$$ (7)

To avoid excessive deviation in the data obtained through expert questionnaires due to subjective bias, it is necessary to conduct a consistency test for all weight values. When the order of the judgment matrix is greater than 2, the consistency index (CI) and the consistency ratio (CR) should be calculated. If CR < 0.1, the judgment matrix is considered to exhibit satisfactory consistency, and its weight allocation can therefore be accepted. Otherwise, the judgment matrix should be adjusted until the consistency requirement is satisfied. CI is calculated as follows:


$$\begin{array}{c} CI=\frac{\lambda_{max}-n}{n-1} \end{array}$$ (8)

$$\begin{array}{c} \lambda_{\text{max}}=\sum_{i=1}^{n}\frac{{\left[IW\right]}_{i}}{{nW}_{i}} \end{array}$$ (9)

Where λ_max_ is the maximum eigenvalue of the judgment matrix.

CR is calculated as follows:


$$\begin{array}{c} CR=\frac{CI}{RI} \end{array}$$ (10)

The random consistency index (RI) values of the judgment matrix are shown in Table 5.

**Table 5:** Average random consistency index RI value of the judgment matrix.

Matrix order	1	2	3	4	5	6	7	8	9	10
RI	0	0	0.52	0.89	1.12	1.26	1.36	1.41	1.46	1.49

#### 3.4.4. Marginal probabilities of root nodes.

In a Bayesian network, the determination of node parameters—that is, conditional probabilities—is a key factor affecting the reliability of the model. Because domain experts often lack a unified probabilistic reference framework for quantitative judgment, cognitive bias and evaluation difficulty may arise during the process of subjective probability assignment, resulting in insufficient consistency of parameter estimates and compromised model accuracy. To address this issue, this study defined expert evaluation intervals based on a probability scale. The expert evaluation intervals are presented in Table 6.

**Table 6:** Expert evaluation range.

Qualitative description	Probability interval
Almost always	0.9 ~ 1
Usually	0.8 ~ 0.9
Frequently	0.7 ~ 0.8
Often	0.6 ~ 0.7
More likely than not	0.5 ~ 0.6
Occasionally	0.4 ~ 0.5
Seldom	0.3 ~ 0.4
Rarely	0.2 ~ 0.3
Very rarely	0.1 ~ 0.2
Almost never	0 ~ 0.1

A questionnaire survey was conducted with u relevant experts to elicit the root-node probabilities of each qualitative indicator. To minimize the influence of individual subjectivity, the Bayesian network parameters were determined by calculating the weighted average of the experts’ evaluation scores in conjunction with their respective weights. Table 7 presents the evaluation indicators and corresponding criteria.

**Table 7:** Expert-qualification indicators and scoring criteria.

Weight indicator	Scoring criterion (points)
Expert title (x_1_)	Above professor level (10); Professor (9); Associate professor (8); Below associate professor level (7)
Research achievements in the field (x_2_)	Outstanding (10); Relatively outstanding (9); Average (8)
Basis for judgment (x_3_)	Investigation/research-based judgment (10); Experience-based judgment (9); Intuition-based judgment (8)
Years of engagement in the research field (x_4_)	More than 10 years (10); More than 5 years and less than 10 years (9); Within 5 years (8)
Confidence in subjective assessment (x_5_)	Very confident (10); Relatively confident (9); Moderately confident (8)

As shown in Table 7, let P (X_ij_ = H | E_i_) denote the probability assigned by the i – th expert to the occurrence of indicator X_ij_, and let W_i_ represent the weight of the i – th expert, subject to the constraint W_1_ + W_2_ + … + W_u_ = 1. Accordingly, the root-node probability can be obtained using the weighted average formula as follows:


$$\begin{array}{c} P\left(X_{ij}=H\right)=\sum_{i=1}^{u}P\left(X_{ij}=H|E_{i}\right)\cdot W_{i} \end{array}$$ (11)

For the calculation of the expert weights W_i_, this study employed the entropy weight method [34]. The entropy weight method requires all data values to be strictly greater than zero. Therefore, if zero values occur after data standardization, a non-negative translation should be applied to the standardized data, for example, by adding a small constant such as 0.01, 0.001, or 0.0001. The procedure for calculating weights using the entropy weight method is as follows:

(1) Standardize the data:

$$\begin{array}{c} {Positive\text{\textasciitilde{}}indicator:a}_{ij}=\frac{x_{ij}-min\left(x_{ij}\right)}{max\left(x_{ij}\right)-min\left(x_{ij}\right)} \end{array}$$ (12)

$$\begin{array}{c} Negative\text{\textasciitilde{}}indicator:\text{\textasciitilde{}}a_{ij}=\frac{max\left(x_{ij}\right)-x_{ij}}{max\left(x_{ij}\right)-min\left(x_{ij}\right)} \end{array}$$ (13)

(2) The proportion of the i – th sample under the j – th indicator is calculated as follows:

$$\begin{array}{c} P_{ij}=\frac{a_{ij}}{\sum_{i=1}^{n}a_{ij}} \end{array}$$ (14)

(3) The entropy value of each indicator is calculated as follows:

$$\begin{array}{c} e_{j}=-\frac{\text{1}}{ln\left(n\right)}\cdot \sum_{i=1}^{n}\left(P_{ij}\cdot ln\left(P_{ij}\right)\right) \end{array}$$ (15)

(4) The weight of each indicator is calculated as follows:

$$\begin{array}{c} W_{j}=\frac{1-e_{j}}{\sum_{j=1}^{m}\left(1-e_{j}\right)} \end{array}$$ (16)

#### 3.4.5. Determination of indicator weights.

Based on the constructed evaluation index system, five experts were invited to assess each indicator in light of their professional knowledge and experience. According to Equation (3), the five judgment matrices were aggregated using the geometric mean method, yielding the integrated matrix for indicators A_1_-A_5_, as presented in Table 8.

**Table 8:** Integrated matrix of A_1_-A_5_.

Indicator	A_1_	A_2_	A_3_	A_4_	A_5_
A_1_	1	0.7579	0.6361	0.9441	0.4671
A_2_	1.3195	1	0.9146	0.2474	0.2971
A_3_	1.572	1.0934	1	0.7677	0.9642
A_4_	1.0592	4.0428	1.3026	1	0.7155
A_5_	2.1409	3.366	1.0371	1.3976	1

According to Equation (5), the rows of the integrated judgment matrix were multiplied to obtain the vector K. According to Equation (6), the n – th root of each component of K was then taken to derive the eigenvector M. Finally, according to Equation (7), the weight vector W for indicators A_1_ - A_5_ was obtained. The resulting K, M and W are presented as follows.


$$\begin{array}{c} K=\left[\begin{array}{c} @c@0.2126\\ 0.0887\\ 1.2723\\ 3.9910\\ 10.4466 \end{array}\right]M=\left[\begin{array}{c} @c@0.7337\\ 0.6160\\ 1.0493\\ 1.3189\\ 1.5988 \end{array}\right]W=\left[\begin{array}{c} @c@0.1380\\ 0.1159\\ 0.1974\\ 0.2481\\ 0.3007 \end{array}\right] \end{array}$$

According to Equations (8)–(10), the CR of the judgment matrix was calculated as 0.0607, which is lower than 0.1. This indicates that the judgment matrix exhibits satisfactory consistency and that its weight allocation is therefore acceptable. The local weights of the criterion-level (A-level) and sub-criterion-level (B-level) indicators are presented in Table 9. The complete weights of the indicator-level nodes are provided in S2 Table.

**Table 9:** Local weights of the criterion-level and sub-criterion-level nodes.

Criterion-level node	Local weight	Local weights of sub-criterion-level nodes
A_1_	0.138	B_1_ = 0.2542; B_2_ = 0.3104; B_3_ = 0.4354
A_2_	0.1159	B_4_ = 0.5140; B_5_ = 0.2486; B_6_ = 0.2374
A_3_	0.1974	B_7_ = 0.6217; B_8_ = 0.3783
A_4_	0.2481	B_9_ = 0.5405; B_10_ = 0.4595
A_5_	0.3007	B_11_ = 0.5746; B_12_ = 0.4254

Taking the target node O as a representative example, A1–A5 are its direct parent nodes. Based on the local weights reported in Table 9, P(O = Y) was calculated for each combination of parent-node states. Because O has five binary parent nodes, there are 32 possible state combinations. Representative entries from the CPT for node O are presented in Table 10, while the complete CPTs are provided in S3 Table.

**Table 10:** Representative entries from the CPT for target node O.

A_1_	A_2_	A_3_	A_4_	A_5_	P(O = Y)
Y	Y	Y	Y	Y	1
Y	Y	Y	Y	N	0.6993
Y	N	N	N	N	0.138
N	Y	N	N	N	0.1159
N	N	Y	N	N	0.1974
N	N	N	Y	N	0.2481
Y	N	Y	N	N	0.3354
N	N	N	N	Y	0.3007
N	N	N	N	N	0

#### 3.4.6. Indicator node probabilities.

The high-quality development level of graduate education was quantified through a questionnaire survey completed by 10 invited experts. To ensure data reliability, sufficient communication was conducted with the experts in advance to ensure that they fully understood the questionnaire and could therefore provide accurate evaluations. The expert scores for the indicators, after standardization and non-negative translation, are presented in Table 11.

**Table 11:** Expert scores for the indicators.

Indicator	E_1_	E_2_	E_3_	E_4_	E_5_	E_6_	E_7_	E_8_	E_9_	E_10_
x_1_	1.001	0.001	0.501	0.001	0.001	0.001	0.001	0.001	0.501	0.001
x_2_	1.001	0.501	0.001	1.001	1.001	1.001	0.501	1.001	0.501	1.001
x_3_	0.001	1.001	0.501	0.001	0.001	1.001	0.501	0.001	0.001	1.001
x_4_	1.001	1.001	0.501	0.001	0.001	1.001	1.001	0.001	0.501	0.001
x_5_	0.001	0.001	0.001	0.001	1.001	1.001	1.001	1.001	1.001	1.001

Using Equations (14)–(16), entropy values and corresponding weights of the expert-qualification indicators were obtained, as presented in Table 12.

**Table 12:** Entropy values and weights of the expert-qualification indicators.

Indicator	E_1_	E_2_	E_3_	E_4_	E_5_	E_6_	E_7_	E_8_	E_9_	E_10_
Entropy	0.6859	0.6594	0.6886	0.0196	0.438	0.8626	0.8278	0.438	0.8297	0.6859
Weight	0.0844	0.0915	0.0457	0.2633	0.1509	0.0369	0.0462	0.1509	0.0457	0.0844

Based on the expert weights and evaluation results, the final prior probabilities of the qualitative root nodes were calculated using Equation (11), as shown in Table 13. The complete prior probabilities for qualitative root nodes E_1_–E_10_ are provided in S4 Table.

**Table 13:** Expert evaluations of qualitative root nodes E_1_–E_10_.

Indicator	C_5_	C_7_	C_11_	C_13_	C_15_	C_17_	C_18_	C_23_	C_25_	C_26_
P(C_i_ = Y)	0.5373	0.544	0.7343	0.6561	0.638	0.7617	0.3709	0.4427	0.5873	0.2971

#### 3.4.7. Data roles and methodological workflow.

The different data sources used in this study served distinct purposes across the stages of indicator identification, structural modeling, and parameter estimation. Policy documents and relevant literature were used to define the research scope and generate the initial set of influencing factors but were not directly involved in the parameterization of the Bayesian network. Multi-stakeholder interview data were analyzed through thematic coding to consolidate the candidate indicators, while the 692 valid questionnaires were used primarily for candidate-indicator screening and structural validation. On this basis, the Delphi method, correlation analysis, and content validation were jointly applied to establish the final set of 26 evaluation indicators. During model construction, 15 experts assessed the direct influence relationships among the indicators to establish the ISM adjacency matrix, and the resulting hierarchical relationships were mapped onto the Bayesian network topology. Pairwise-comparison judgments from five experts were analyzed using AHP to calculate the local weights of indicators at different levels, which subsequently provided the weighting basis for constructing the conditional probability tables of non-root nodes. The prior probabilities of quantitative root nodes were obtained by mapping observed indicator values onto probability intervals, whereas those of qualitative root nodes were calculated by aggregating the probabilistic assessments of 10 experts using expert weights derived through the entropy weighting method. The network topology, conditional probability tables, and prior probabilities of the root nodes were then entered into the model for forward inference, diagnostic inference, and sensitivity analysis. The composition of the participant and expert groups is reported in S5 Table, and the expert-selection criteria are provided in S6 Table.

The methods employed in this study were not simply combined in parallel; rather, they formed a sequential analytical framework tailored to the requirements of the different research stages. Specifically, ISM was used to identify the hierarchical relationships and directed dependency structure among the indicators. AHP was applied to determine their relative importance at different levels on the basis of expert judgments and to provide the weighting basis for the Bayesian network CPTs. The entropy weighting method was used to account for differences in expert-qualification indicators and to reduce subjectivity in the determination of expert weights. Finally, the Bayesian network integrated the structural relationships identified through ISM with the estimated probability parameters to support forward inference, diagnostic inference, and sensitivity analysis under uncertainty. Accordingly, the methods served distinct functions in indicator validation, structural identification, parameter determination, and probabilistic reasoning, with the output of each stage providing input for the subsequent stage.

## 4. Result

### 4.1. Forward inference

Forward inference refers to the process of deriving the probability distributions of other variables on basis of known variables. In a Bayesian network, the occurrence probability of the event represented by each child node is inferred through the layer-by-layer propagation of joint probabilities under different node-state combinations, ultimately yielding the probability of the terminal nodes. In the present study, the probability of the high-quality development of graduate education in mining engineering was predicted on basis of the occurrence probabilities of the nodes corresponding to each indicator. After the marginal probabilities of the root nodes were entered, the network was updated, thereby completing the calculation of the prior probabilities of all nodes and producing the probability distributions of each node, as shown in Fig 3.

**Fig 3:**
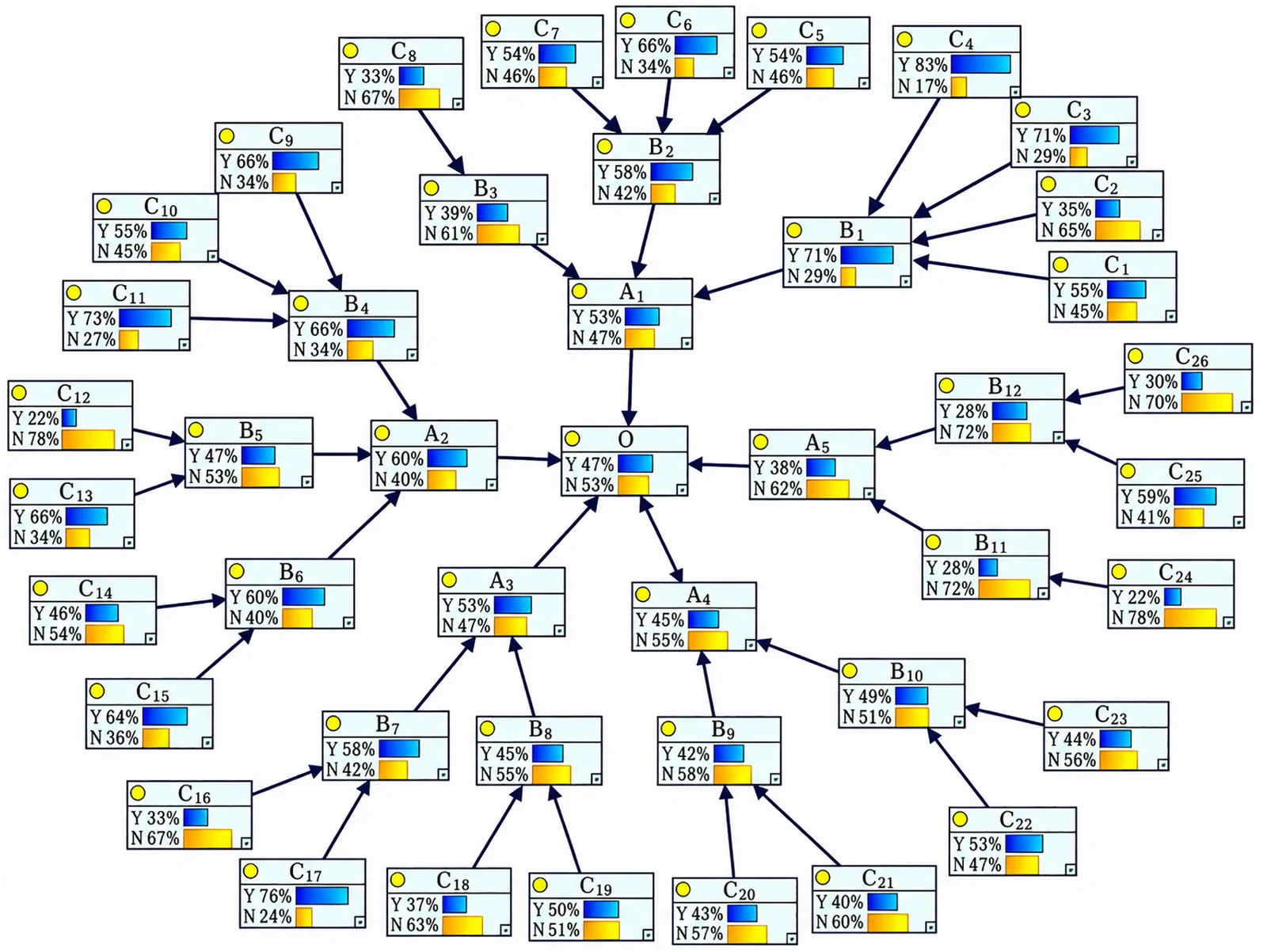
Forward inference model of the Bayesian network.

According to the Bayesian network results, the probability of the high-quality development of graduate education in mining engineering (O) is 47%, which, according to Table 3, corresponds to Level 2, indicating an overall medium level of development. At the criterion level, Training foundation (A_1_), Support capacity (A_2_), Process control (A_3_), Outcome transformation (A_4_), and Industry adaptability (A_5_) are all classified as Level 2, suggesting a generally moderate level of development across the five dimensions. Among them, Support capacity (A_2_) shows the highest probability (53%), whereas Industry adaptability (A_5_) exhibits the lowest probability (38%). At the sub-criterion level, a certain degree of variation is observed among the indicators. Curriculum quality (B_1_) has the highest probability (71%) and is classified as Level 1, indicating a relatively high level of development. In contrast, Job adaptability (B_11_) has the lowest probability (28%) and is classified as Level 3, indicating a low level of development. The remaining sub-criteria are generally at a medium level of development.

To examine the influence of alternative state-conversion rules on the model results, different threshold schemes were applied to convert node probabilities into binary Y/N states for robustness analysis. Thresholds of p = 0.45、0.50 and 0.55 were considered. When the positive-state probability of a node satisfied P (C_i_ = Y) ≥ p, the node was assigned state Y; otherwise, it was assigned state N. The Bayesian network was evaluated under each threshold scheme, and changes in the positive-state probability of the target node O were compared, as shown in Table 14.

**Table 14:** Alternative discretization-threshold analysis.

Scheme	Threshold p	State-conversion rule	P(O = Y)	Development category
Baseline	—	Original probabilistic states	47.00%	Medium
1	0.45	Y if P(C_i_ = Y) ≥ 0.45, otherwise N	45.80%	Medium
2	0.5	Y if P(C_i_ = Y) ≥ 0.50, otherwise N	46.70%	Medium
3	0.55	Y if P(C_i_ = Y) ≥ 0.55, otherwise N	44.90%	Medium

As shown in Table 14, when different discretization thresholds were used to convert node probabilities into binary states, the positive-state probability of target node O remained between 44.9% and 47.0%. The observed variation can be attributed primarily to the conversion of the original probabilistic information into deterministic binary states, which inevitably results in some loss of uncertainty information. Nevertheless, the development category of the target node remained medium under all threshold schemes, indicating that the model results were reasonably robust to alternative node-state classification rules.

### 4.2. Diagnostic inference

Diagnostic inference refers to the process of inferring the underlying causes of an observed outcome. In a Bayesian network, once the probability of occurrence of the leaf node has been determined, the posterior probability distributions of the corresponding influencing-factor nodes can be inferred in sequence. On this basis, the combination of factors most likely to induce the target event can be identified according to the magnitude of the posterior probabilities. In the present study, diagnostic inference was performed on the established Bayesian network model for the high-quality development of graduate education in mining engineering to obtain the posterior probabilities of the relevant nodes and thereby identify the principal factors leading to the occurrence of the target node state. Specifically, the node “High-quality development level of graduate education in mining engineering” was selected as the evidence node, and its state “Y” was set to 100%, yielding the results shown in Fig 4.

**Fig 4:**
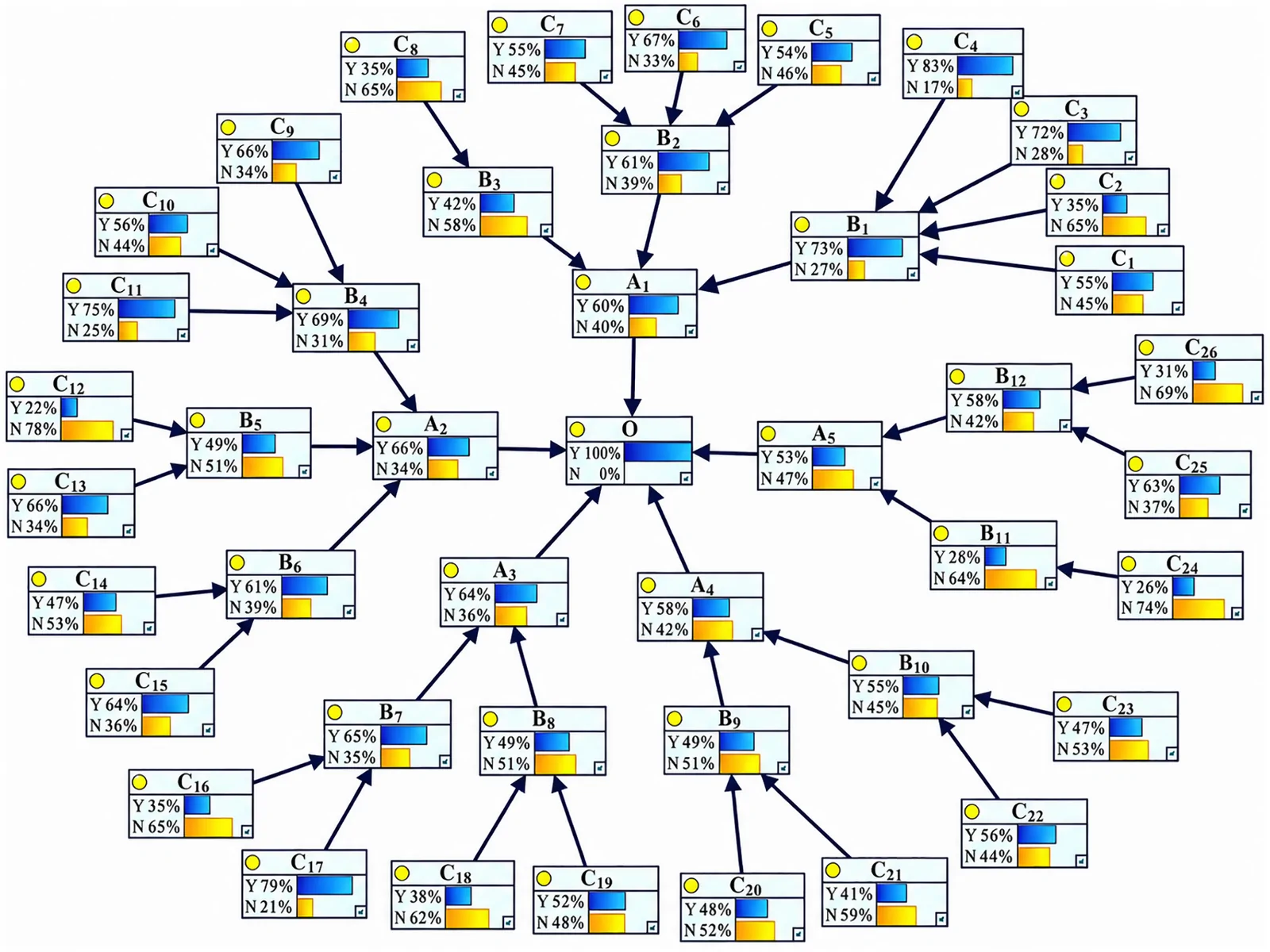
Diagnostic inference for mining graduate education development.

As shown in Fig 4, when the target node O was instantiated in state Y, the key indicators exhibited substantial differences in their posterior positive-state probabilities. The core curriculum renewal cycle (C_4_) reached 83%. The duration of field practice (C_6_), supervisors’ technological updating capability (C_11_), thesis-process control (C_17_), and interdisciplinary course coverage (C_3_) also exhibited comparatively high posterior probabilities, indicating that these indicators were closely associated with the positive state of the target node within the model. By contrast, the positive-state probability of research platform support was only 22%, substantially lower than those of the other indicators. This result suggests that research platform support represents a relatively weak component of the current graduate education system and warrants further verification and attention in subsequent evaluation and improvement efforts. Strengthening research and innovation platforms and improving access to research resources may therefore constitute an important direction for enhancing graduate education in mining engineering. In addition, training foundation (A_1_), which had the largest number of parent nodes, was selected as an evidence node, and the posterior probabilities of the related nodes were inferred, as shown in Fig 5.

**Fig 5:**
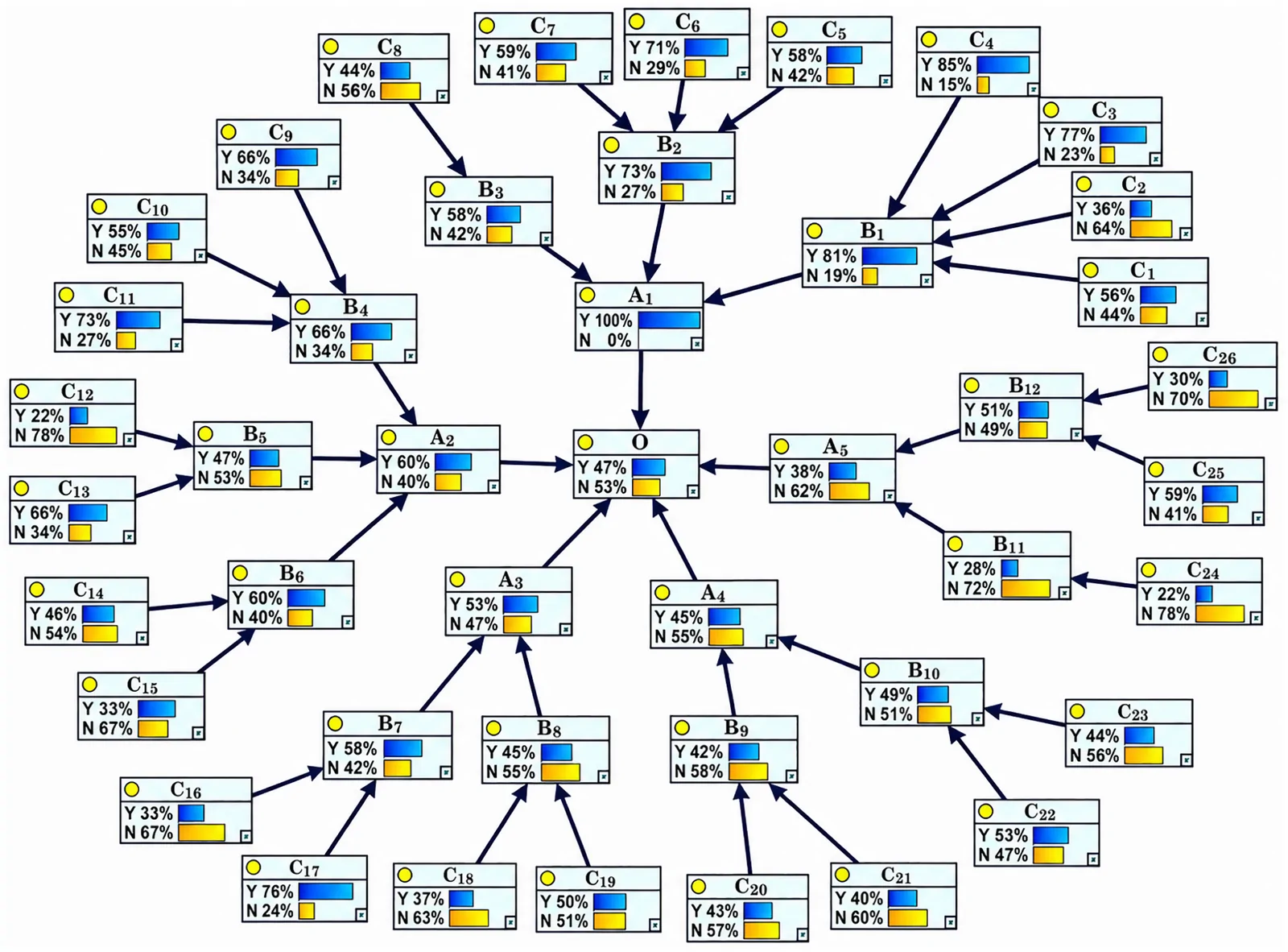
Diagnostic inference for the training foundation.

As shown in Fig 5, the Core curriculum renewal cycle (C_4_) exhibits the highest development probability, reaching 85%, indicating that this indicator provides particularly strong support for Training foundation. In contrast, the development probability of the Proportion of green mining courses (C_2_) is only 36%, suggesting that it remains insufficient to meet the demands of development in the new era. Under the current policy and industrial context, green development has become a central direction for the transformation and upgrading of the geoscience and mining sector, as well as an essential requirement for implementing the national ecological strategy. Accordingly, integrating green development into graduate education should not be understood as a simple increase in the number of relevant courses; rather, it entails a profound transformation in which the educational content, industrial development needs, and national strategic priorities are brought into closer alignment.

### 4.3. Sensitivity analysis

Sensitivity analysis is used to examine how small variations in model numerical parameters—specifically, prior probabilities and conditional probabilities—affect output parameters, such as posterior probabilities, thereby identifying the variables that exert the most influence on the target node. The GeNIe software implements the algorithm proposed by Kjaerulff and van der Gaag, which, given a set of target nodes, efficiently computes the full set of derivatives of the target node’s posterior probability distribution with respect to each numerical parameter in the Bayesian network. In this study, the sensitivity analysis module of GeNIe was employed with the “high-quality development level of graduate education in mining engineering” set as the target node. The results of the sensitivity analysis are shown in Fig 6, and the corresponding sensitivity tornado diagram is presented in Fig 7.

**Fig 6:**
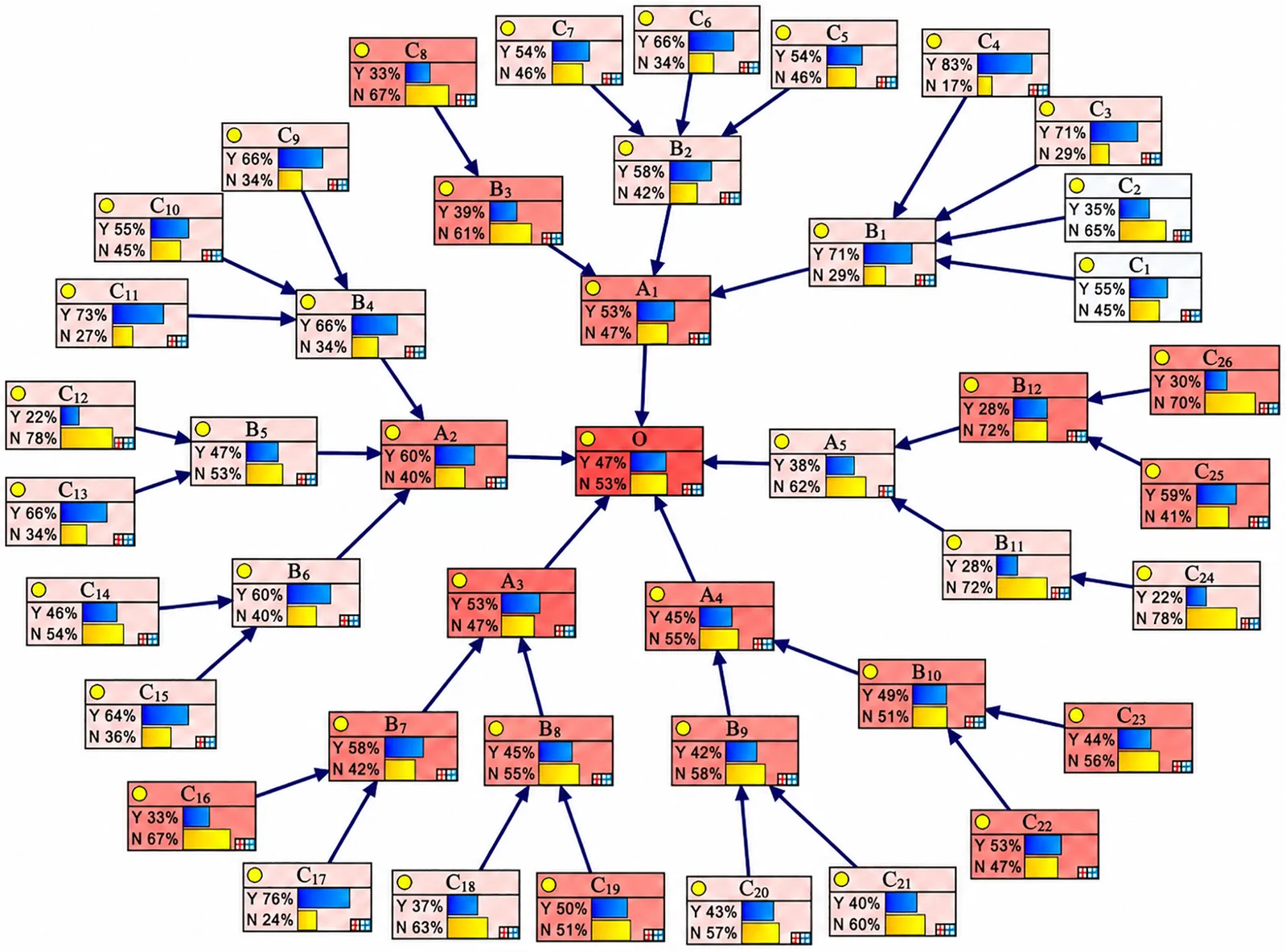
Sensitivity analysis of mining graduate education development.

**Fig 7:**
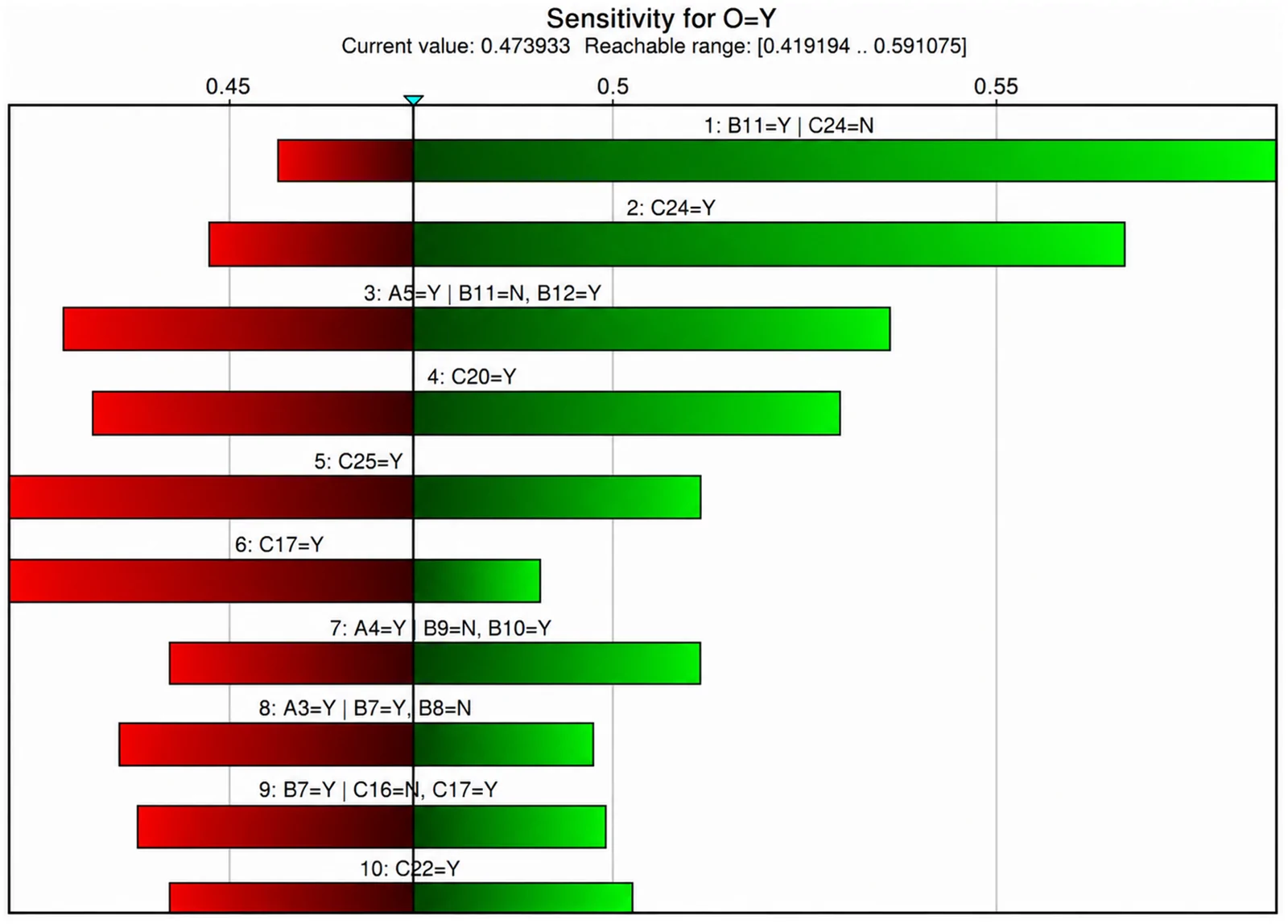
Sensitivity tornado of mining graduate education development.

As shown in Fig 6, the sensitivity intensity of the root nodes can be divided into three levels. The high-sensitivity nodes (first level), depicted with the darkest colors, include C_16_ (Supervision frequency in practical training), C_17_ (Thesis-process control), C_18_ (Response speed of enterprise feedback), C_19_ (Participation of industry experts in review), C_20_ (Proportion of high-level papers), C_21_ (Proportion of high-quality granted invention patents), C_22_ (Thesis-outcome transformation rate), C_23_ (Value of technology transfer), C_24_ (Employment rate of graduates in relevant mining-industry positions), C_25_ (Speed of training-program adjustment), and C_26_ (Enrollment scale of specialized training tracks). The low-sensitivity nodes (third level), shown with lighter colors, are C_1_ (Proportion of intelligent geoscience-and-mining courses) and C_2_ (Proportion of green mining courses), while all remaining nodes fall into the medium-sensitivity category (second level).

Fig 7 presents the results of the sensitivity analysis for the target variable (O = Y) in the Bayesian network model. The green bars indicate factors that exert a positive influence on the occurrence of the target event, while the red bars indicate factors that have a negative influence. Longer green bars reflect factors with a strong positive driving effect on the target event, whereas longer red bars indicate factors that, in their current state, significantly reduce the probability of the target event occurring. When the probability of high-quality development in graduate education in mining engineering is P(O = Y), the tornado diagram indicates that the conditional probability P(B_11_ = Y|C_24_ = N) exhibits the highest sensitivity.

## 5. Discussion

### 5.1. Key findings regarding the high-quality development of graduate education in mining engineering

The results indicate that curriculum quality, practical training, supervisors’ technological updating capability, process control, and industry adaptability are closely associated with the overall quality assessment, whereas research platform support, outcome transformation capacity, and the alignment between the training system and the evolving needs of industrial transformation remain comparatively weak. The forward-inference results showed that support capacity had the highest positive-state probability, while industry adaptability had the lowest. This pattern not only reflects uneven development across the five criterion-level dimensions but also reveals a structural feature of graduate education in mining engineering: relatively strong internal support but insufficient external adaptability.

The comparatively high probability of support capacity may be attributed to the combined role of faculty capacity, research platforms, management systems, and incentive mechanisms, which together form the institutional and resource foundation of the graduate training process. Compared with instructional activities that affect only specific stages of training, supervisors’ professional competence, platform and equipment conditions, training regulations, and incentive mechanisms can simultaneously influence curriculum renewal, research training, thesis supervision, practical training, and academic or applied outcomes, thereby providing broad-based support across the system. Previous studies have shown that high-quality doctoral training depends not only on academically competent supervisors but also on research infrastructure, financial support, academic networks, and timely and effective supervision. Supervisors also play an important role in translating institutional policies and training requirements into concrete research activities [35]. However, the diagnostic-inference results identified research platform support as a clear weakness. This suggests that the relatively high overall probability of support capacity arose primarily from the combined contribution of faculty competence, institutional arrangements, and incentive mechanisms rather than from uniformly strong performance across all of its components. By contrast, industry adaptability had the lowest positive-state probability, suggesting a gap between the pace of adjustment in graduate training and the speed of technological transformation in the mining industry. As the industry moves toward intelligent, green, digital, and low-carbon development, employers increasingly require digital competence, interdisciplinary knowledge, and engineering-practice capabilities. However, curriculum renewal, program revision, and the development of practical training platforms often lag behind these emerging demands. Engineering education research has consistently emphasized competency-oriented reform, interdisciplinary training, practice-based learning, and university–industry collaboration. Studies of the mining workforce have likewise suggested that future mining work will become increasingly digitalized, automated, and remotely operated, with competency requirements shifting toward hybrid capabilities that combine technical knowledge, field experience, and digital skills, while placing greater emphasis on continuous learning and multi-stakeholder collaboration [36,37]. Therefore, the relatively low probability of industry adaptability should not be interpreted solely as an employment-outcome issue. Rather, it suggests that the training program, curriculum content, and practical training system have not yet established a sufficiently timely and stable mechanism for dynamic alignment with emerging industrial needs. At the same time, regional industrial structures, enterprise recruitment cycles, and fluctuations in occupational demand may also affect indicators related to industry adaptability; therefore, this result should not be attributed entirely to the graduate training system itself.

Compared with existing research, the findings of this study show consistency at three levels. First, graduate education research has emphasized faculty competence, research resources, and institutional support as essential foundations for high-quality research training, which is consistent with the comparatively high probability of support capacity identified in this study. Second, engineering education research has highlighted competency-oriented curriculum reform, interdisciplinary training, practical learning, and university–industry collaboration, echoing the importance of curriculum quality, practical training, and enterprise feedback identified here. Third, research on mining-workforce development has shown that intelligent and green transformation is reshaping mining occupations and their associated competency requirements, providing an industry-level explanation for why industry adaptability emerged as a comparatively weak dimension.

### 5.2. Policy and managerial implications

Based on the findings of this study, reform of graduate education in mining engineering should focus on improving the conversion of internal support conditions into stronger training outcomes and greater industry adaptability. Policy priorities should gradually shift from expanding the quantity of resources toward improving platform utilization efficiency, training-process quality, and responsiveness to industry needs.

First, the practical contribution of research-platform resources to graduate training should be strengthened. Although support capacity performed comparatively well overall, research platform support remained a relatively weak component within this dimension. Universities and education authorities should therefore avoid evaluating platform development solely on the basis of laboratory space, the number of instruments, or the scale of financial investment. Greater attention should be given to the degree of platform openness and resource sharing, equipment-utilization efficiency, and the actual contribution of platforms to research innovation and engineering practice. Universities and enterprises should also jointly establish research and practical training platforms in frontier areas such as intelligent mining, green mining, mine safety, disaster early warning, and ecological restoration, thereby promoting the coordinated allocation of equipment, engineering scenarios, and research tasks. Second, a dynamic mechanism for adjusting training programs in response to industry needs should be established. Given the comparatively weak performance of industry adaptability and job adaptability, universities should incorporate technological changes in the mining industry, enterprise demand for talent, graduates’ workplace performance, and employer feedback into the revision of training programs. Curriculum systems and training programs should not be updated solely according to fixed review cycles; rather, they should be revised in response to actual developments in intelligent, digital, green, and low-carbon transformation. Third, curriculum renewal, practical training, and thesis-process control should be treated as priority areas for improving training quality. Universities should incorporate emerging technologies, updated standards, and new engineering challenges in mining into core courses to prevent curriculum content from becoming disconnected from industrial development. Practical training should include a greater proportion of real mining-engineering projects, enterprise-based tasks, and interdisciplinary problems, enabling graduate students to integrate disciplinary knowledge, digital technologies, and engineering methods in complex settings. At the same time, quality control throughout the thesis process—from topic selection and proposal review to mid-term assessment and final evaluation—should be progressively strengthened. Particular attention should be paid to the alignment of thesis topics with industry needs, disciplinary frontiers, and practical engineering problems. In addition, differentiated training policies should receive greater attention. Academic-degree students should place greater emphasis on theoretical innovation, original research, and interdisciplinary research capabilities, whereas professional-degree students should focus more strongly on engineering practice, technology application, and the ability to address complex field-based problems. Differentiated requirements should therefore be established for curriculum structure, duration of practical training, supervisor allocation, and outcome evaluation, rather than applying an identical training model to all students. Finally, a collaborative governance mechanism involving government agencies, universities, enterprises, and industry organizations should be developed. Government agencies can provide policy support through platform development, targeted investment, and quality-evaluation systems. Universities should assume responsibility for curriculum reform, supervisor development, and training-process management. Enterprises and industry organizations should provide authentic engineering contexts and opportunities for developing job-related competencies. Together, these actors can establish a dynamic cycle of “industry-needs identification–training-program adjustment–training-process monitoring–outcome evaluation–feedback-based readjustment,” thereby converting existing strengths in faculty, institutions, and platforms into stronger research-innovation capacity, engineering-practice capability, and industry adaptability among graduate students. From the perspective of implementation feasibility, management-oriented measures—such as annual curriculum updating, enterprise-demand feedback, thesis-topic review, and the open sharing of existing platforms—can be prioritized in the short term. By contrast, major research-platform construction and large-scale equipment renewal are more appropriately treated as medium- to long-term investment priorities.

### 5.3. Limitations and future research

By integrating ISM with a Bayesian network, this study developed a multilevel evaluation and probabilistic reasoning framework for the high-quality development of graduate education in mining engineering. The framework identified the hierarchical structure among the relevant factors and used forward inference, diagnostic inference, and sensitivity analysis to reveal key dimensions, comparatively weak components, and potential priorities for intervention. Nevertheless, several limitations should be acknowledged.

(1) the screening of evaluation indicators, the hierarchical relationships identified through ISM, the indicator weights, and some conditional probability parameters depended to a certain extent on expert knowledge. Because the high-quality development of graduate education in mining engineering involves complex factors related to training processes, industry adaptability, and graduate competencies, large-scale longitudinal data covering the complete training process remain limited. Expert-informed methods were therefore used to parameterize the Bayesian network. Although this study sought to reduce potential bias arising from individual judgments through multi-stakeholder interviews, the Delphi method, AHP consistency testing, multi-expert aggregation, and entropy-based expert weighting, the resulting probabilities essentially represent structured judgments informed by the theoretical framework and domain knowledge rather than predictive probabilities learned from large-scale historical data. Accordingly, the Bayesian network developed in this study is more appropriately regarded as an expert-informed decision-support tool under uncertainty. It can be used to identify key factors, examine potential influence pathways, and compare development probabilities across alternative scenarios. Future research could incorporate longitudinal follow-up data, graduate training-process records, and graduate employment data to support data-driven parameter learning and dynamic updating, thereby improving the empirical calibration, predictive performance, and validity of the model.
(2) The participants and experts were drawn primarily from Chinese universities specializing in geoscience and mining and from related industry organizations. The applicability of the proposed framework to other countries, regions, or engineering disciplines therefore remains to be established, and the findings should not be generalized beyond the present context without further validation. Future studies could conduct cross-regional, cross-institutional, and interdisciplinary comparisons to examine whether the importance of the identified nodes and their structural relationships remains consistent across different educational systems and industrial contexts.
(3) Both the quality of graduate education in mining engineering and industry demand for professional competencies evolve over time. As intelligent mining, green and low-carbon technologies, and industrial policies continue to develop, node probabilities and their conditional relationships may also change. Future research could therefore establish a continuously updated data-collection mechanism incorporating annual information on curriculum revision, research-platform development, enterprise feedback, and graduates’ job performance. These data could be used to construct a dynamic Bayesian network capable of monitoring changes in training quality over time and evaluating the effects of policy and managerial interventions.

## 6. Conclusion

(1) This study proposed an ISM-based evaluation index system and a Bayesian network evaluation model for the high-quality development of graduate education in mining engineering. It improves the existing evaluation framework, fills a gap in the industry-specific evaluation of graduate education in mining engineering, and provides a feasible theoretical and methodological framework for promoting its high-quality development.
(2) Through ISM-based hierarchical analysis and Bayesian network modeling, this study identified the factors associated with the high-quality development of graduate education in mining engineering, including curriculum quality, practical training, faculty capacity, research platforms, and industry adaptability. These factors play different roles and occupy different hierarchical positions in the improvement of educational quality.
(3) The Bayesian network sensitivity analysis showed that the probability of the target node was relatively sensitive to the conditional probability parameters between job adaptability and the employment rate of graduates in relevant mining-industry positions. This finding suggests that education administrators should give priority to optimizing these key areas to promote the overall improvement of educational quality.

## Supporting information

S1 Table Evaluation index system for the high-quality development of graduate education in mining engineering. (DOCX)

S2 Table Complete indicator weights. (DOCX)

S3 Table Conditional probability table for target node O. (DOCX)

S4 Table Expert evaluations of E_1_–E_10_. (DOCX)

S5 Table Composition of research samples and expert panels. (DOCX)

S6 Table Specific selection criteria, response rates, and conflict-of-interest assessment. (DOCX)

S7 Table Codes and definitions of the 26 indicators. (DOCX)

S8 Table Expert-judgment rules and ISM coding procedure. (DOCX)

S9 Table Adjacency matrix A. (DOCX)

S10 Table Reachability matrix M. (DOCX)

S11 Table ISM hierarchical partitioning procedure. (DOCX)
