# Supplementary material for: Evaluation of the high-quality development of graduate education in mining engineering based on ISM and Bayesian networks
Source: PLoS One. 2026 Oct 1;21(10):e0359427. doi: 10.1371/journal.pone.0359427 (PMC13630315; doi:10.1371/journal.pone.0359427)
Supplement: Supplementary file 1 [file pone.0359427.s001.docx]

**S1 Table. Evaluation index system for the high-quality development of graduate education in mining engineering**

| Target Level (O) | Criterion Level (A, 5 items) | Sub-criterion Level (B, 12 items) | Indicator Level (C, 26 items) | Indicator Type |
| --- | --- | --- | --- | --- |
| High-quality development level of graduate education in mining engineering | A_1_: Training foundation | B_1_: Curriculum quality | C_1_: Proportion of intelligent geoscience-and-mining courses | Foundational support indicator |
|  |  |  | C_2_: Proportion of green mining courses | Foundational support indicator |
|  |  |  | C_3_: Interdisciplinary course coverage | Foundational support indicator |
|  |  |  | C_4_: Core curriculum renewal cycle | Foundational support indicator |
|  |  | B_2_: Practical training | C_5_: Quality level of practical training bases | Foundational support indicator |
|  |  |  | C_6_: Duration of field practice | Practical support indicator |
|  |  |  | C_7_: Mine-safety practice coverage | Practical support indicator |
|  |  | B_3_: Thesis quality | C_8_: Alignment of thesis topics with industry needs | Process control indicator |
|  | A_2_: Support capacity | B_4_: Faculty capacity | C_9_: Dual-supervisor allocation rate | Practical support indicator |
|  |  |  | C_10_: Supervisors’ industry-practice experience | Foundational support indicator |
|  |  |  | C_11_: Supervisors’ technological updating capability | Core driving indicator |
|  |  | B_5_: Platform resources | C_12_: Research platform support | Core driving indicator |
|  |  |  | C_13_: Platform equipment adaptability | Core driving indicator |
|  |  | B_6_: Management system | C_14_: Coverage of flexible training systems | Core driving indicator |
|  |  |  | C_15_: Strength of reward incentives | Core driving indicator |
|  | A_3_: Process control | B_7_: Process monitoring | C_16_: Supervision frequency in practical training | Process control indicator |
|  |  |  | C_17_: Thesis-process control | Process control indicator |
|  |  | B_8_: Feedback mechanism | C_18_: Response speed of enterprise feedback | Process control indicator |
|  |  |  | C_19_: Participation of industry experts in review | Practical support indicator |
|  | A_4_: Outcome transformation | B_9_: Academic outcomes | C_20_: Proportion of high-level papers | Intermediate outcome indicator |
|  |  |  | C_21_: Proportion of high-quality granted invention patents | Intermediate outcome indicator |
|  |  | B_10_: Applied outcomes | C_22_: Thesis-outcome transformation rate | Intermediate outcome indicator |
|  |  |  | C_23_: Value of technology transfer | Development outcome indicator |
|  | A_5_: Industry adaptability | B_11_: Job adaptability | C_24_: Employment rate of graduates in relevant mining-industry positions | Development outcome indicator |
|  |  | B_12_: Responsiveness to industry needs | C_25_: Speed of training-program adjustment | Development outcome indicator |
|  |  |  | C_26_: Enrollment scale of specialized training tracks | Development outcome indicator |
