# Supplementary material for: Evaluation of the high-quality development of graduate education in mining engineering based on ISM and Bayesian networks
Source: PLoS One. 2026 Oct 1;21(10):e0359427. doi: 10.1371/journal.pone.0359427 (PMC13630315; doi:10.1371/journal.pone.0359427)
Supplement: Supplementary file 2 [file pone.0359427.s002.docx]

**S2 Table. Complete indicator weights**

| Indicators | Weight | Indicators | Weight | Indicators | Weight |
| --- | --- | --- | --- | --- | --- |
| A_1_ | 0.1380 | B_1_ | 0.2542 | C_1_ | 0.0605 |
|  |  |  |  | C_2_ | 0.0738 |
|  |  |  |  | C_3_ | 0.5690 |
|  |  |  |  | C_4_ | 0.2968 |
|  |  | B_2_ | 0.3104 | C_5_ | 0.3215 |
|  |  |  |  | C_6_ | 0.3677 |
|  |  |  |  | C_7_ | 0.3107 |
|  |  | B_3_ | 0.4354 | C_8_ | 1.0000 |
| A_2_ | 0.1159 | B_4_ | 0.5140 | C_9_ | 0.1763 |
|  |  |  |  | C_10_ | 0.3422 |
|  |  |  |  | C_11_ | 0.4815 |
|  |  | B_5_ | 0.2486 | C_12_ | 0.4260 |
|  |  |  |  | C_13_ | 0.5740 |
|  |  | B_6_ | 0.2374 | C_14_ | 0.6185 |
|  |  |  |  | C_15_ | 0.3815 |
| A_3_ | 0.1974 | B_7_ | 0.6217 | C_16_ | 0.4145 |
|  |  |  |  | C_17_ | 0.5855 |
|  |  | B_8_ | 0.3783 | C_18_ | 0.3545 |
|  |  |  |  | C_19_ | 0.6455 |
| A_4_ | 0.2481 | B_9_ | 0.5405 | C_20_ | 0.7386 |
|  |  |  |  | C_21_ | 0.2614 |
|  |  | B_10_ | 0.4595 | C_22_ | 0.5346 |
|  |  |  |  | C_23_ | 0.4654 |
| A_5_ | 0.3007 | B_11_ | 0.5746 | C_24_ | 1.0000 |
|  |  | B_12_ | 0.4254 | C_25_ | 0.7273 |
|  |  |  |  | C_26_ | 0.2727 |
