# Supplementary material for: Evaluation of the high-quality development of graduate education in mining engineering based on ISM and Bayesian networks
Source: PLoS One. 2026 Oct 1;21(10):e0359427. doi: 10.1371/journal.pone.0359427 (PMC13630315; doi:10.1371/journal.pone.0359427)
Supplement: Supplementary file 3 [file pone.0359427.s003.docx]

**S3 Table. Conditional probability table for target node O**

| A_1_ | A_2_ | A_3_ | A_4_ | A_5_ | P(O=Y) |
| --- | --- | --- | --- | --- | --- |
| Y | Y | Y | Y | Y | 1.0000 |
| Y | Y | Y | Y | N | 0.6993 |
| Y | Y | Y | N | Y | 0.7519 |
| Y | Y | Y | N | N | 0.4512 |
| Y | Y | N | Y | Y | 0.8026 |
| Y | Y | N | Y | N | 0.5019 |
| Y | Y | N | N | Y | 0.5546 |
| Y | Y | N | N | N | 0.2539 |
| Y | N | Y | Y | Y | 0.8841 |
| Y | N | Y | Y | N | 0.5834 |
| Y | N | Y | N | Y | 0.6361 |
| Y | N | Y | N | N | 0.3354 |
| Y | N | N | Y | Y | 0.6868 |
| Y | N | N | Y | N | 0.3861 |
| Y | N | N | N | Y | 0.4387 |
| Y | N | N | N | N | 0.1380 |
| N | Y | Y | Y | Y | 0.8620 |
| N | Y | Y | Y | N | 0.5613 |
| N | Y | Y | N | Y | 0.6139 |
| N | Y | Y | N | N | 0.3132 |
| N | Y | N | Y | Y | 0.6646 |
| N | Y | N | Y | N | 0.3639 |
| N | Y | N | N | Y | 0.4166 |
| N | Y | N | N | N | 0.1159 |
| N | N | Y | Y | Y | 0.7461 |
| N | N | Y | Y | N | 0.4454 |
| N | N | Y | N | Y | 0.4981 |
| N | N | Y | N | N | 0.1974 |
| N | N | N | Y | Y | 0.5488 |
| N | N | N | Y | N | 0.2481 |
| N | N | N | N | Y | 0.3007 |
| N | N | N | N | N | 0.0000 |
