# Supplementary material for: Evaluation of the high-quality development of graduate education in mining engineering based on ISM and Bayesian networks
Source: PLoS One. 2026 Oct 1;21(10):e0359427. doi: 10.1371/journal.pone.0359427 (PMC13630315; doi:10.1371/journal.pone.0359427)
Supplement: Supplementary file 4 [file pone.0359427.s004.docx]

**S4 Table. Expert evaluations of E_1_–E_10_**

| Indicator | C_5_ | C_7_ | C_11_ | C_13_ | C_15_ | C_17_ | C_18_ | C_23_ | C_25_ | C_26_ |
| --- | --- | --- | --- | --- | --- | --- | --- | --- | --- | --- |
| E_1_ | 0.65 | 0.55 | 0.70 | 0.65 | 0.65 | 0.80 | 0.40 | 0.35 | 0.40 | 0.35 |
| E_2_ | 0.45 | 0.60 | 0.75 | 0.70 | 0.70 | 0.75 | 0.45 | 0.45 | 0.50 | 0.30 |
| E_3_ | 0.60 | 0.50 | 0.70 | 0.60 | 0.70 | 0.75 | 0.30 | 0.40 | 0.55 | 0.25 |
| E_4_ | 0.50 | 0.60 | 0.80 | 0.75 | 0.60 | 0.85 | 0.35 | 0.50 | 0.60 | 0.30 |
| E_5_ | 0.55 | 0.55 | 0.65 | 0.60 | 0.65 | 0.70 | 0.40 | 0.45 | 0.65 | 0.25 |
| E_6_ | 0.45 | 0.65 | 0.65 | 0.65 | 0.70 | 0.80 | 0.45 | 0.35 | 0.55 | 0.40 |
| E_7_ | 0.40 | 0.55 | 0.70 | 0.55 | 0.75 | 0.75 | 0.25 | 0.55 | 0.70 | 0.35 |
| E_8_ | 0.60 | 0.45 | 0.80 | 0.65 | 0.60 | 0.65 | 0.30 | 0.45 | 0.65 | 0.25 |
| E_9_ | 0.50 | 0.70 | 0.65 | 0.70 | 0.55 | 0.70 | 0.40 | 0.40 | 0.55 | 0.30 |
| E_10_ | 0.60 | 0.35 | 0.70 | 0.50 | 0.65 | 0.80 | 0.45 | 0.35 | 0.60 | 0.35 |
