# Supplementary material for: Evaluation of the high-quality development of graduate education in mining engineering based on ISM and Bayesian networks
Source: PLoS One. 2026 Oct 1;21(10):e0359427. doi: 10.1371/journal.pone.0359427 (PMC13630315; doi:10.1371/journal.pone.0359427)
Supplement: Supplementary file 5 [file pone.0359427.s005.docx]

**S5 Table. Composition of research samples and expert panels**

| Research stage | Sample size | Composition of participants | Relationship among samples and use of data |
| --- | --- | --- | --- |
| Multi-stakeholder interviews | 63 participants | 20 doctoral supervisors from geoscience- and mining-related disciplines, 10 graduate education administrators, 15 technical professionals from mining enterprises, 8 enterprise human-resource managers, 6 experts from the China National Coal Association, and 4 representatives from the Henan Coal Society | Used for indicator identification. Some participants were subsequently included in the expert pool; however, interview data and subsequent evaluation data were collected and used separately. |
| Candidate-indicator questionnaire screening | 692 valid responses | Graduate education-related participants from 28 universities offering geoscience- and mining-related programs, including graduate students, supervisors, and graduate education administrators | An independent questionnaire sample used to reduce 68 candidate indicators to 32 indicators and support subsequent correlation analysis. These data were not combined with expert evaluations and were not used for Bayesian network parameter learning. |
| Indicator validation and Delphi consultation | 20 experts | 10 doctoral supervisors from geoscience- and mining-related disciplines, 6 technical professionals from mining enterprises, and 4 graduate education administrators | A repeated consultation panel consisting of the same expert group across three rounds. Used to optimize the 32 indicators into 28 indicators. This group partially overlapped with subsequent task-specific expert groups. |
| ISM structural relationship assessment | 15 experts | 5 heads of geoscience- and mining-related disciplines, 5 senior technical managers from mining enterprises, and 5 educational evaluation experts | A structural-judgment expert group. It partially overlapped with the Delphi, AHP, and probability-assessment expert groups; however, an independent questionnaire was used, and the results were applied only to the adjacency matrix, ISM hierarchical analysis, and network topology construction. |
| AHP indicator weighting | 5 experts | Selected from the expert pool consisting of university mining/geoscience supervisors, mining enterprise technical professionals, and education-management/evaluation experts | A weighting task group. Members could partially overlap with other expert groups, but the pairwise-comparison matrices were completed independently. The results were used to calculate local weights and construct conditional probability tables. |
| Qualitative root-node probability assessment | 10 experts | Experts with experience in mining engineering education, industry practice, or educational evaluation | A probability-assessment task group. It partially overlapped with other expert groups, but probability questionnaires were conducted independently. The results were used to estimate prior probabilities of qualitative root nodes. |
