# Supplementary material for: Evaluation of the high-quality development of graduate education in mining engineering based on ISM and Bayesian networks
Source: PLoS One. 2026 Oct 1;21(10):e0359427. doi: 10.1371/journal.pone.0359427 (PMC13630315; doi:10.1371/journal.pone.0359427)
Supplement: Supplementary file 6 [file pone.0359427.s006.docx]

**S6 Table. Specific selection criteria, Response rates, and Conflict-of-interest assessment**

| Research stage | Selection criteria | Response rate | Conflict-of-interest assessment |
| --- | --- | --- | --- |
| Multi-stakeholder interviews | Purposive sampling was adopted. Participants were selected on the basis of their familiarity with graduate training, technical and employment needs in mining enterprises, or the requirements of mining-industry transformation. | 100% | No separate conflict-of-interest declaration was collected from interview participants. |
| Candidate-indicator questionnaire screening | Participants were drawn from universities offering geoscience- and mining-related programs and were able to evaluate the importance, disciplinary relevance, and operational feasibility of the candidate indicators. | 86.5 | Not applicable |
| Indicator validation and Delphi consultation | Experts had experience in graduate training in mining engineering, technical practice in mining enterprises, or educational administration; were familiar with relevant quality requirements. | 80% | No separate standardized conflict-of-interest declaration was administered; questionnaires were completed independently in each round. |
| ISM structural relationship assessment | Experts were familiar with mining engineering training systems, industrial technology needs, or educational evaluation and were able to assess the direct influence relationships among the 26 indicators. | 90% | No separate standardized conflict-of-interest declaration was administered; an independent judgment questionnaire was used. |
| AHP indicator weighting | Experts possessed relevant disciplinary knowledge and practical experience, were familiar with the evaluation index system and its hierarchical relationships, and were able to complete pairwise comparisons. | 100% | No separate standardized conflict-of-interest declaration was administered; an independent judgment questionnaire was used. |
| Qualitative root-node probability assessment | Experts were familiar with graduate training in mining engineering and the qualitative indicators and were able to provide independent evaluations using the predefined probability intervals | 95% | No separate standardized conflict-of-interest declaration was administered; evaluation results were submitted independently. |
