# Supplementary material for: Evaluation of the high-quality development of graduate education in mining engineering based on ISM and Bayesian networks
Source: PLoS One. 2026 Oct 1;21(10):e0359427. doi: 10.1371/journal.pone.0359427 (PMC13630315; doi:10.1371/journal.pone.0359427)
Supplement: Supplementary file 7 [file pone.0359427.s007.docx]

**S7 Table. Codes and definitions of the 26 indicators**

| Code | Indicator | Indicator type |
| --- | --- | --- |
| C_1_ | Proportion of intelligent geoscience-and-mining courses | Foundational support indicator |
| C_2_ | Proportion of green mining courses | Foundational support indicator |
| C_3_ | Interdisciplinary course coverage | Foundational support indicator |
| C_4_ | Core curriculum renewal cycle | Foundational support indicator |
| C_5_ | Quality level of practical training bases | Foundational support indicator |
| C_6_ | Duration of field practice | Practical support indicator |
| C_7_ | Mine-safety practice coverage | Practical support indicator |
| C_8_ | Alignment of thesis topics with industry needs | Process control indicator |
| C_9_ | Dual-supervisor allocation rate | Practical support indicator |
| C_10_ | Supervisors’ industry-practice experience | Foundational support indicator |
| C_11_ | Supervisors’ technological updating capability | Core driving indicator |
| C_12_ | Research platform support | Core driving indicator |
| C_13_ | Platform equipment adaptability | Core driving indicator |
| C_14_ | Coverage of flexible training systems | Core driving indicator |
| C_15_ | Strength of reward incentives | Core driving indicator |
| C_16_ | Supervision frequency in practical training | Process control indicator |
| C_17_ | Thesis-process control | Process control indicator |
| C_18_ | Response speed of enterprise feedback | Process control indicator |
| C_19_ | Participation of industry experts in review | Practical support indicator |
| C_20_ | Proportion of high-level papers | Intermediate outcome indicator |
| C_21_ | Proportion of high-quality granted invention patents | Intermediate outcome indicator |
| C_22_ | Thesis-outcome transformation rate | Intermediate outcome indicator |
| C_23_ | Value of technology transfer | Development outcome indicator |
| C_24_ | Employment rate of graduates in relevant mining-industry positions | Development outcome indicator |
| C_25_ | Speed of training-program adjustment | Development outcome indicator |
| C_26_ | Enrollment scale of specialized training tracks | Development outcome indicator |
