# Supplementary material for: Evaluation of the high-quality development of graduate education in mining engineering based on ISM and Bayesian networks
Source: PLoS One. 2026 Oct 1;21(10):e0359427. doi: 10.1371/journal.pone.0359427 (PMC13630315; doi:10.1371/journal.pone.0359427)
Supplement: Supplementary file 8 [file pone.0359427.s008.docx]

**S8 Table. Expert-judgment rules and ISM coding procedure**

| Item | Description |
| --- | --- |
| Composition of the expert panel | The assessment was conducted by 15 experts, including heads of geoscience- and mining-related disciplines, senior technical professionals from mining enterprises, and experts in graduate education evaluation. |
| Object of judgment | The experts determined whether a direct influence relationship existed between each ordered pair of indicators C_i_ and C_j_. |
| Direction of influence | The relationship C_i_ → C_j_ indicates that, in the formation process of the high-quality development of graduate education, Ci exerts a direct facilitating, constraining, or conditional influence on C_j_. |
| Binary coding rule | If Ci was judged to have a direct influence on C_j_, the corresponding element a_ij_ in the adjacency matrix was coded as 1; otherwise, it was coded as 0. |
| Resolution of expert disagreement | Direct influence relationships were determined through consensus among the experts. When disagreements arose, the experts discussed them with reference to the indicator definitions and the logic of high-quality development until consensus was reached. |
| Adjacency matrix | The adjacency matrix A is a 26 × 26 binary matrix, in which the row indicator Ci represents the influencing factor and the column indicator, C_j_ represents the influenced factor. |
| Reachability matrix | Based on the adjacency matrix A，the reachability matrix was calculated using M = (A + I) ^k^ where I is the identity matrix and k is the number of iterations. |
| Transitivity check | The reachability matrix satisfies (A + I)^k^ = (A + I) (k + 1) |
| Hierarchical partitioning rule | For each indicator C_i_, the reachability set R(C_i_), antecedent set Q(C_i_), and intersection R(C_i_) ∩ Q(C_i_) were calculated. If R(C_i_) ∩ Q(C_i_) = R(C_i_), then C_i_ was assigned to the current highest level. The indicators assigned to that level were then removed, and the procedure was repeated until all indicators had been hierarchically partitioned. |
