# Supplementary material for: Evaluation of the high-quality development of graduate education in mining engineering based on ISM and Bayesian networks
Source: PLoS One. 2026 Oct 1;21(10):e0359427. doi: 10.1371/journal.pone.0359427 (PMC13630315; doi:10.1371/journal.pone.0359427)
Supplement: Supplementary file 11 [file pone.0359427.s011.docx]

**S11 Table. ISM hierarchical partitioning procedure**

| Iteration | Indicators included in the current partitioning round | Indicators satisfying R(C*_i_*)∩Q(C*_i_*)=R(C*_i_*) | Partitioning result |
| --- | --- | --- | --- |
| Round 1 | C_1_–C_26_ | C_23_、C_24_、C_26_ | Level 1 |
| Round 2 | C_1_–C_22_、C_25_ | C_20_、C_21_、C_22_、C_25_ | Level 2 |
| Round 3 | C_1_–C_19_ | C_8_、C_16_、C_17_、C_18_ | Level 3 |
| Round 4 | C_1_–C_7_、C_9_–C_15_、C_19_ | C_6_、C_7_、C_9_、C_19_ | Level 4 |
| Round 5 | C_1_–C_5_、C_10_–C_15_ | C_1_、C_2_、C_3_、C_4_、C_5_、C_10_ | Level 5 |
| Round 6 | C_11_–C_15_ | C_11_、C_12_、C_13_、C_14_、C_15_ | Level 6 |
